# Recent Advances in Sensing Materials Targeting Clinical Volatile Organic Compound (VOC) Biomarkers: A Review

**DOI:** 10.3390/bios13010114

**Published:** 2023-01-09

**Authors:** Akhilesh Kumar Pathak, Kankan Swargiary, Nuntaporn Kongsawang, Pannathorn Jitpratak, Noppasin Ajchareeyasoontorn, Jade Udomkittivorakul, Charusluk Viphavakit

**Affiliations:** 1 International School of Engineering (ISE), Intelligent Control Automation of Process Systems Research Unit, Chulalongkorn University, Bangkok 10330, Thailand; 2 Biomedical Engineering Program, Faculty of Engineering, Chulalongkorn University, Bangkok 10330, Thailand

**Keywords:** breath analysis, sensor, volatile organic compound, biomarker, molecularly imprinted polymer, metal oxides, composites, graphene, gas sensor

## Abstract

In general, volatile organic compounds (VOCs) have a high vapor pressure at room temperature (RT). It has been reported that all humans generate unique VOC profiles in their exhaled breath which can be utilized as biomarkers to diagnose disease conditions. The VOCs available in exhaled human breath are the products of metabolic activity in the body and, therefore, any changes in its control level can be utilized to diagnose specific diseases. More than 1000 VOCs have been identified in exhaled human breath along with the respiratory droplets which provide rich information on overall health conditions. This provides great potential as a biomarker for a disease that can be sampled non-invasively from exhaled breath with breath biopsy. However, it is still a great challenge to develop a quick responsive, highly selective, and sensitive VOC-sensing system. The VOC sensors are usually coated with various sensing materials to achieve target-specific detection and real-time monitoring of the VOC molecules in the exhaled breath. These VOC-sensing materials have been the subject of huge interest and extensive research has been done in developing various sensing tools based on electrochemical, chemoresistive, and optical methods. The target-sensitive material with excellent sensing performance and capturing of the VOC molecules can be achieved by optimizing the materials, methods, and its thickness. This review paper extensively provides a detailed literature survey on various non-biological VOC-sensing materials including metal oxides, polymers, composites, and other novel materials. Furthermore, this review provides the associated limitations of each material and a summary table comparing the performance of various sensing materials to give a better insight to the readers.

## 1. Introduction

Nowadays, exhaled breath analysis is considered an important area of research in order to diagnose several diseases at an early stage. In ancient times, several physicians observed certain odors in the human exhaled breath which are associated with a certain specific disease and its stage. For example, a diabetic patient was observed to exhale a ‘fruity’ odor while renal failure has shown an association with some ‘fishy’ smell [1]. Later, in modern science, these exhaled odors were recognized as volatile organic compounds (VOCs) [2,3]. These VOCs are produced in the human body either from exogenous VOCs or endogenous VOCs. The exogenous VOCs contain the compounds inhaled from the outside environment, which are produced following the oral ingestion of food and compounds obtained from smoking cigarettes. On the other hand, endogenous VOCs are compounds made from symbiotic bacteria and/or blood-borne compounds which can be released into the environment via the lungs. Therefore, considering the potential of VOCs being used as biomarkers, their real-time monitoring and analysis are essential and recognized as a new frontier in health inspections and disease diagnostics [4,5].

The concentrations of available VOCs in the exhaled breath are determined in the range of nanomolar to picomolar levels. Hence, it is always a challenging task to distinguish normal individuals from the patients suffering from some chronic disease via endogenously formed VOCs. However, collecting the exhaled breath samples is painless, extremely simple, and noninvasive which provides a great benefit to the patients. Therefore, it is worth investigating human breath VOC detection. Gas chromatography–mass spectrometry (GC–MS) has been considered as the most accurate, selective, and sensitive method which has been utilized for several years to detect these VOCs from exhaled breath. The GC–MS technique utilizes the ion-pair extraction of the breath sample and quantification by mass spectrometry to monitor VOC concentrations as low as part per trillion (ppt). Although the instrument is promising, it faces the most significant limitations due to its cost, time consumption, off-site analyses, and the requirement for skilled technicians. To overcome this limitation, several researchers have proposed various user-friendly interfaces for the sensing system. To detect these VOC biomarkers in the exhaled human breath such as exploiting catalyst [6], electrochemical [7], resistance [8], and optical sensors [9], coating the novel-sensing materials on these devices is the common practice to enhance and improve the sensing performance.

**Figure 1:**
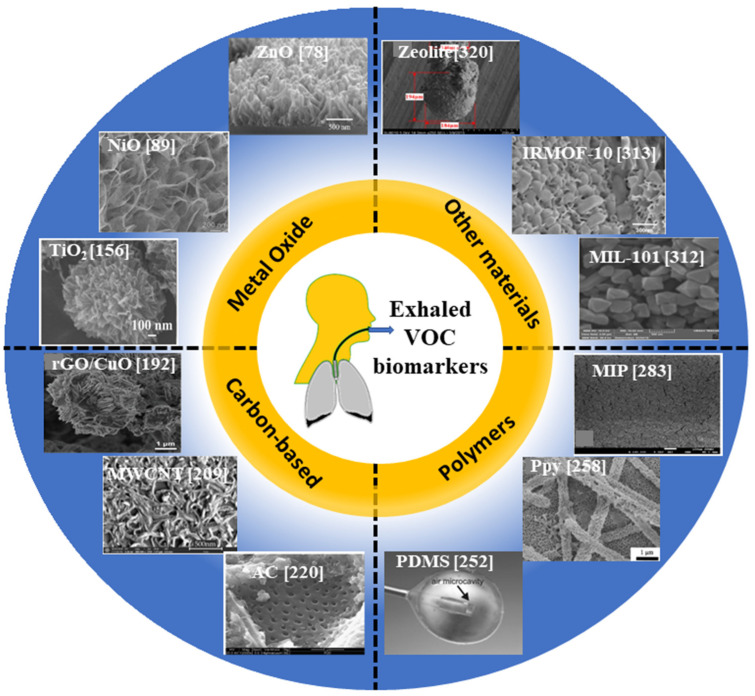
A summary of the sensing materials used for detecting VOC biomarkers.

Nanoscale sensing materials of sizes varying in the range of 2 nm to 100 nm (within the Debye length of the surface) are utilized as the highly sensitive sensing elements in vapor/gas sensors due to their large surface-to-volume-ratio and their unique optical, chemical, and electrical properties compared to the non-nanoscale sensing materials [10]. These vapor/gas-sensitive materials usually include metal oxide, carbon-based materials, and polymers with various morphological structures, integrated over conventional transducers (e.g., optical, capacitive, resistive, and gravimetric). The sensing properties of these materials depend on the chemisorption of negatively charged oxygen adsorbents in the air [11]. Gas chemisorption is an energy-activated process and the oxygen species require different temperatures to take place on the surface of grains. In air, the oxygen molecules capture electrons from the materials and then exist as O_2_^−^ (<100 °C), O^−^ (100 °C to 300 °C), and O^2−^ (>300 °C) on the surface of sensing materials [11,12]. Generally, the relatively high temperature may endow adsorbed VOC gaseous molecules with high activation and could speed up the rate of gas diffusion. It is conducive to the occurrence of chemical reactions between the oxygen species and the target VOC molecules [13,14]. It also depends on the chemical reaction between gas/vapor and sensing material, or the diffusion of VOC into the bulk of the sensing material. Therefore, an intensive study to employ these sensing materials for vapor/gas sensing systems is required. In this review, the non-biological materials are mainly considered and discussed for the VOC sensors. With these materials, a sensor can achieve rapid response, simple synthesis, and good thermal and chemical stabilities. In addition, the associated disadvantages, and challenges of these materials in terms of the sensitivity, response time, detection limit, selectivity, and their corresponding sensing mechanisms are also presented and discussed. For instance, a summary of VOC’s sensing materials is shown in Figure 1.

This review is organized in the following manner: Section 2 contains information on the main sources of VOCs in the human body, while Section 3 provides a brief discussion on major VOCs present in the exhaled breath and the associated diseases/disorders. In Section 4, we discussed various classical approaches, that have been utilized for several years to detect VOCs in human breath. In Section 5, Section 6, Section 7 and Section 8 we comprehensively provide a literature review on various sensing materials including metal oxide (zinc oxide, molybdenum trioxide, titanium dioxide), carbon-based materials (graphene, multi-wall carbon nanotubes, activated carbon), polymers (polydimethylsiloxane, polypyrrole, polythiophene), and other materials (MIL-n series, Irmofs series, Zeolites), respectively. Section 9 and Section 10 include the future prospects and clinical challenges along with a comparative analysis between the gold standard and the other material-based devices, respectively. Finally, an overall conclusion is drawn in Section 11.

## 2. Origins of VOCs in the Human Body

The VOC composition in the exhaled breath is complex and determined by several external and internal factors, e.g., metabolic, inflammatory, and oxidative stress processes and environmental conditions [15]. These VOCs originate from several sources, including the respiratory tract, tissues, organs, and microbiomes. The source of VOCs can be categorized into two types, (i) exogenous and (ii) endogenous. The exogenous VOCs are defined as the VOCs resulting from external exposure. Exogenous VOCs comprise a wide range of sources and have largely been overlooked as environmental contaminants in biological samples. Nowadays, exogenous VOCs gain huge interest as most of them interact with biological systems and provide valuable information relevant to health and diseases. Whereas, endogenous VOCs are produced throughout the body and are selected and distributed in the bloodstream. From the blood, these VOCs are exchanged into air in the lungs and are then exhaled in respiratory droplets and atmospheric gases. Exhaled VOCs provide a source of useful biomarkers with clear associations with the body’s metabolism. Breath Biopsy enables non-invasive collection and analysis of the VOC biomarkers from breath. Usually, it takes one minute for blood to flow around the entire circulatory system. By sampling the breath for a minute or longer, even at low levels of the VOC biomarkers from all parts of the body, it can be pre-concentrated, detected, and identified. It is easy to increase the sensitivity to aid in detecting subtle changes during the very early stages of the disease by simply extending the sample collection time [16]. In the next section, we provide detailed information on various VOCs and the associated disorders.

## 3. Diseases and Disorders Indicated by Important Biomarkers

The most important VOC biomarkers of the diseases in the human body are isoprene (C_5_H_8_), acetone (C_3_H_6_O), ammonia (NH_3_), methane (CH_4_), aldehyde (R−CH=O), and hydrogen sulfide (H_2_S) [17]. This section will briefly summarize the metabolic processes that extract these VOC biomarkers and outline the disorders which are indicated by the amount that is present in the exhaled breath. The detailed information on these VOCs is listed below [18]:

**(i). Isoprene:** Isoprene generally appears copiously in the exhaled breath of patients suffering from some chronic diseases. In 2001, McGrath et al. investigated the level of isoprene in exhaled breath of patients suffering from heart failure [19]. Breath isoprene production in subjects with chronic heart failure was significantly reduced in the patients from 83 pmol/min·kg compared to the controls which are 168 pmol/ min·kg. Later in 2009, Amal et al. diagnosed the stage of lung cancer by investigating the concentration of isoprene in the exhaled breath [20]. According to the report, the person affected with lung cancer exhaled a lower concentration of isoprene, acetone, and methanol compared to the healthy control. In 2019, Phillip et al. performed a historical study of 53 pediatric Type-I diabetes mellitus (T1DM) patients in parallel to the children suffering from chronic kidney disease and healthy control [21]. The outcome suggested that the T1DM patients exhaled significantly higher concentrations of ethanol, isoprene, and pentanal compared to the control one. Additionally, the alveolar concentrations of isopropanol and pentanal were significantly different in patients with adequate and poor long-term metabolic control. From the report, it was observed that isoprene is linked to the biosynthesis of cholesterol and is most probably stored in fat tissue.

**(ii). Acetone:** Acetone is a metabolite of glucose and human fat. It appears as the most common VOC in exhaled human breath. It was, firstly, recognized as the diabetes biomarker by Petters et al. in 1857 [22]. Glucose has been popularly known to be a primary source of energy in the human body. The insulin, which is a hormone, generated by the pancreas enables cells to absorb glucose molecules. When the pancreas cannot generate insulin, it is considered T1DM. On the other hand, if the pancreas can generate insulin but fails to be used by the body, it is known as Type-II diabetes mellitus (T2DM). Usually, the body can extract energy from glucose by breaking body fat to produce energy [18]. This process is called ketogenesis. Ketogenesis is the source of all ketone bodies including acetone in humans. All ketone substances, including acetone, are produced during ketogenesis in the human body. The concentration of acetone in the exhaled human breath is directly associated with the concentration level of blood sugar. The acetone level in exhaled breath and blood sugar has a strong linear correlation, which is utilized to diagnose the stage of diabetes [23]. The higher concentration of the exhaled breath acetone indicates the severity of diabetes and hence can be an alarming situation. For a non-diabetic person, the exhaled breath acetone level is ≤0.9 parts per million (ppm). For the moderately diabetic person, the acetone level is 0.9 ppm to 1.8 ppm, and for the severe-stage diabetic people this value can be several tens of ppm.

**(iii). Ammonia:** Ammonia plays a vital role in nutritional benefits including the synthesis of amino sugar, the synthesis of pyrimidines and purines, producing non-essential amino acids, and maintaining the acid-base balance in the human body. However, when the ammonia concentration exceeds its control level, it becomes toxic to the human body. Therefore, this excess amount of ammonia has to be released from the body by ornithine or the urea cycle which converts this excess ammonia into urea and excretes it in the form of urine via the kidney [24]. This conversion cycle takes place in the kidney and liver. Therefore, any problem associated with kidney and liver functioning will lead to an excess amount of ammonia concentration in the exhaled breath. Hence, the high concentration of ammonia in the exhaled breath indicates several diseases such as liver dysfunction, kidney failure, hepatic encephalopathy, type-II Alzheimer, swelling of the brain, etc. [25,26].

**(iv). Methane:** Methane is considered to be an important gas appearing in exhaled human breath. It is produced by the methane-producing bacteria present in the intestine under anaerobic circumstances [27]. Methane is not present in the breath under normal conditions, but when it is when excessively created. The variation in methane levels can also be observed under other conditions including inflammatory bowel disease, obesity, irritable bowel syndrome, etc. [28]. Methane is produced and can be considered a biomarker for oxidative stress, heart disease, breast cancer, hepatic disease. Therefore, hydrocarbons, including methane, pentane, and ethane, can serve as potential biomarkers for several diseases that involve oxidative stress such as neck and head cancer [29].

**(v). Aldehydes:** Aldehydes, such as hydroxy alkenals, alkenals, and dialdehydes, are the products of lipid peroxidation and alcohol oxidation. Their elevated levels were observed in cancer patients [30]. During lipid peroxidation, polyunsaturated fatty acids interact with free radicals and produce aldehydes. Patients suffering from oxidative stress, liver cancer/disease, and Wilson’s disease have been observed to show a higher concentration of aldehydes in their exhaled breath and/or blood [31]. In several metabolic or genetic disorders such as aging, diabetes, Parkinson’s and Alzheimer’s disease, elevated levels of aldehydes (especially formaldehyde, glyoxal) in blood, urine, and breath have been reported [32].

**(vi). Hydrogen sulfide:** Hydrogen sulfide is a popular VOC with a malodor. It is a significant gasotransmitter in animals and humans signaling several physical processes including neuromodulation, inflammation, cytoprotection, apoptosis, and vascular tone regulation [33,34]. Hydrogen sulfide in the exhaled breath is considered to be the potential biomarker for airway inflammation, asthma, and also dental and oral health [35,36,37].

In Table 1 we summarize all these VOCs along with their molecular formula and associated disorders.

## 4. Techniques of Detecting VOC Biomarkers in the Exhaled Breath

The aforementioned discussions show that the human breath contains a variety of VOCs that serve as biomarkers for various diseases and metabolic problems. Therefore, the real-time monitoring of such VOCs in the exhaled human breath is highly essential to enable non-invasive illness detection. In the following subsections, a detailed discussion has been carried out to understand various techniques developed to detect VOCs in very low concentrations of part per million volumes (ppmv), part per billion volumes (ppbv), and part per trillion volumes (pptv).

### 4.1. Gas Chromatography–Mass Spectrometry (GC–MS) Techniques

**Figure 2:**
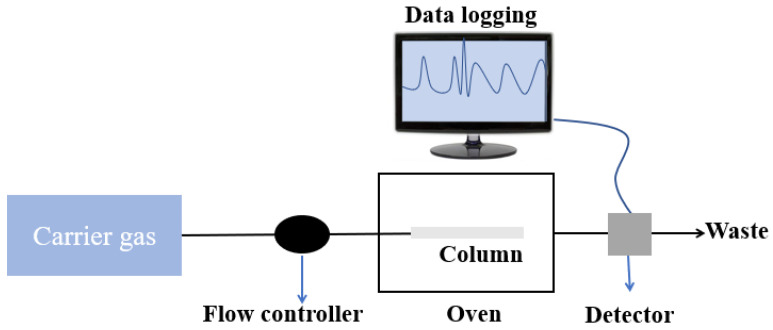
Block schematic diagram of a GC–MS.

GC–MS is a technique in which a mixture of molecules of various compounds travel by a carrier gas (normally helium) via a column that separates molecules and detects them by a detector. In the past decades, this technique has been utilized widely along with various types of detectors to detect VOCs in exhaled breath for the purpose of health monitoring. A block schematic diagram of GC–MS is shown in Figure 2. In 2003, Sanchez et al. detected 25 VOCs in human exhaled breath using a series couple column which includes some of the important biomarkers such as ethanol, methanol, acetone, isoprene, pentane, etc. [54]. The limit of detection (LOD) was observed to be 1–5 ppb in 0.8 L of the exhaled breath. Lord et al. also developed a GC–MS-based analytical detection system which could selectively detect acetone and ethanol in the exhaled breath and reduce the moisture effect to a large extent [55]. Later, Giardina et al. also proposed a low-temperature glassy carbon-based solid-phase mass extraction microfiber which was capable of extracting at least five types of cancer related to VOC biomarkers from the simulated breath. The extracted sample was analyzed using GC–MS with good sensitivity [56]. Lamote et al. utilized e-Nose and GC–MS to distinguish between malignant pleural mesothelioma patients and asymptomatic asbestos-exposed people at a risk of the mentioned disease. Schnabel et al. utilized GC–Time of flight–mass spectrometry (GC–TOF–MS) to demonstrate the non-invasive monitoring of ventilator associated pneumonia in ICU patients by exhaled breath analysis [57]. They identified nearly 12 VOC biomarkers for this purpose. GC–MS was also utilized by Acevedo et al. for gastric cancer [58]. The study proposed the approach to distinguish between healthy people and patients suffering from gastric cancer by the exhaled breath analysis. From all this literature, we can say that GC–MS is considered a potential technique for quantitative analysis for non-invasive detection.

### 4.2. Selected-Ion Flow-Tube Mass Spectrometry (SIFT-MS)

**Figure 3:**
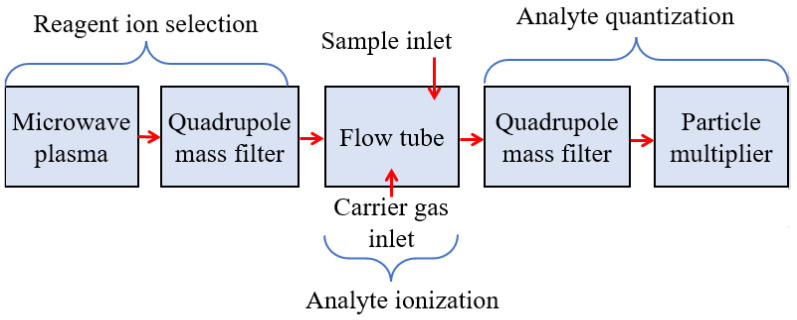
Block schematic diagram of a SIFT-MS.

SIFT-MS is a tool of analytical chemistry which is similar to gas chromatography for the quantitative monitoring of VOCs [59]. In this technique, the VOC samples are ionized by the reagent ions, such as NO^+^, H_3_O^+^, O^2+^, etc., which can be later analyzed by a quadrupole mass spectrometer. Figure 3 shows the block diagram of SIFT-MS. SIFT-MS was first reported to (i) determine the VOC trace presented in the exhaled human breath for the prognosis of the disease and (ii) understand pathophysiological and physiological conditions. In 1996, Smith et al. used SIFT-MS to detect ammonia from the exhaled breath of a known Helicobacter pyroli-infected person which was observed to be increased by ∼4 ppm after an oral dose of 2 g nonradioactive urea [60]. In 1999, Spanel et al. utilized SIFT-MS and O^2+^ as reagent ions in order to quantitatively detect isoprene in the exhaled breath [61]. Later in 2002, Diskin et al. investigated the variation in concentration of common breath VOC biomarkers, such as ammonia, isoprene, ethanol, acetaldehyde, and acetone over a period of 30 days using SIFT-MS with healthy individuals [62]. In the same year, Abbott et al. utilized SIFT-MS to detect acetonitrile in the breath and urinary samples of several smokers and nonsmokers [63]. The result exhibited that the acetonitrile concentration in the exhaled breath was achieved within the range of 17 ppb to 124 ppb, while the urinary acetonitrile concentrations were obtained within the range of 0 μg/L to 150 μg/L that were close to the concentrations previously determined in the blood. Other researchers also utilized SIFT-MS to determine the concentration of various VOCs and diagnosed the stage of related disorders [64,65,66].

### 4.3. Proton-Transfer-Reaction Mass Spectrometry (PTR-MS)

**Figure 4:**
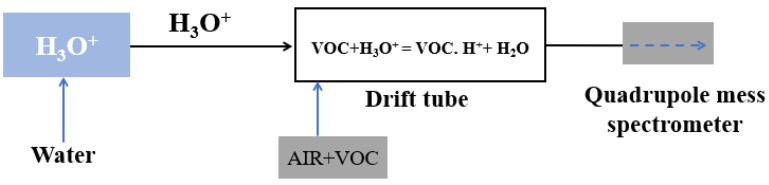
Block schematic diagram of a PTR-MS.

Classical PTR-MS is a tool of analytical chemistry which utilizes gas-phase hydronium ion as n ion with purity >99.5% of source reagent. The PTR-MS is utilized to monitor the absolute concentration of the selected VOCs as low as pptv without any calibration [67]. Figure 4 illustrates the block schematic diagram of PTR-MS. PTR-MS exhibits excellent sensitivity and selectivity which play a crucial role in the exhaled breath analysis to monitor the pathophysiological and physiological state of the human subjects. In 2004, Amann et al. utilized PTR-MS to study the variation in the concentration of different VOCs exhaled in patients during sleep with carbohydrate malabsorption, and inter- and intra-subject variability of a certain mass [68]. Kar et al. utilized PTR-MS to detect cholesterologenesis by investigating the level of isoprene in the exhaled breath [69]. Later, Schmutzhard et al. reported the potential of PTR-MS to diagnose neck and head squamous cell carcinoma by monitoring isoprene in the exhaled breath [70]. Overall, this technique has been exploited widely to detect various VOCs and hence diagnosed the stages of corresponding diseases.

### 4.4. Advantages and Limitations of Classical VOC Detection Techniques

These techniques utilize the ion-pair extraction of the analytes and quantification by mass spectrometry to detect the VOC concentrations. However, a limitation of these techniques is that they can be time-consuming and expensive. They also require a skilled technician to operate which can be done only for off-site analysis. A detailed discussion of the advantages and limitations of these techniques is provided in Table 2.

## 5. Metal Oxides (MOs)

Oxide is a classification of chemical compound that has one or more oxygen atoms including other elements in its composition such as CO_2_, H_2_O, etc. MOs are chemical compounds that are formed by metal and oxygen. In general, MOs are known for their active selective material for sensing applications that encounters optical, chemical, and electronic inducement for analyte molecules detection [71,72,73].

### 5.1. Zinc Oxide (ZnO)

MO nanostructures, such as ZnO, are n-type semiconductors that have a wide band gap energy (3.37 ev at 300 K), large excitation binding energy (60 meV) at RT [74], and a high surface-to-volume ratio, making it a promising candidate for VOC and gas-sensing detection by significantly improving its response [75]. ZnO has been extensively used as an active sensing material as it provides simple synthesis preparation, good mechanical stability, biocompatibility, and offers an admissible response to a different variety of VOCs [76]. Many researchers have reported and studied various forms of ZnO nanostructures, i.e., nanoparticles, nanorods, nanowires, nanosheets, nanotubes, thin films, and 3D hierarchical structures including their application in VOCs vapor detection [74,77,78]. These various structures can be prepared by well-known techniques such as radio frequency sputtering [75,79], hydrothermal [78,80,81], sol-gel coating [82], and solvothermal combined with calcination [74]. The sensing mechanism of the ZnO-based gas sensor is mainly based on the adsorption/desorption of the VOC and gas species onto the oxide active sites and the electronic effects that are produced by the contact resistance of the modified ZnO gas sensors with the noble metals [83].

**Figure 5:**
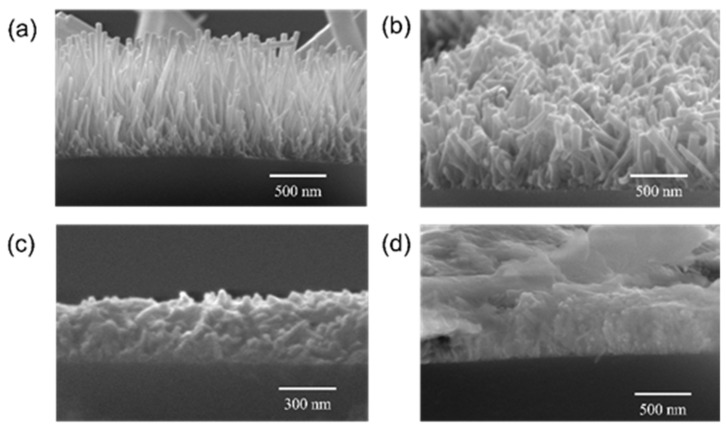
Scanning electron microscope (SEM) micrographs of ZnO nanorods on CSF with growth times of (**a**) 3 h; (**b**) 4 h; (**c**) 5 h; and (**d**) 7 h. (Reprinted/adapted from [78]).

Among the different VOCs, acetone and ethanol are most studied and reported to be detected by different ZnO nanostructures while inadequate numbers of studies are done for other VOCs such as acetylene, n-butanol, cyclohexane, and benzene. Hardan et al. investigated the use of ZnO thin films on thermally oxidized Si substrates to detect isopropanol, acetone, and ethanol and reported the highest sensitivity of acetone detection at around 500 ppm at 400 °C [75]. The mechanism was based on an ionosorption model where the change of the resistance of the metal oxide semiconductor (MOS) in the presence of the test gas was outlined. Bora et al. studied and investigated the ZnO nanorods coated on multimode optical fiber for different chemicals such as ethanol, methanol, toluene, and benzene for its vapor sensing and reported the ethanol average sensitivity to be 22.2% at 50 ppm [84]. The study offered a simple and cost-effective optical sensor utilizing a light scattering from ZnO nanorod structures with a specific length, diameter, and surface coverage of the nanorods. In 2016, Muthukrishnan et al. reported an alternate sol-gel dip coating approach to obtain ZnO thin film to reduce the cost and complications [82]. They investigated the sensing response of acetone, ethanol, and acetaldehyde gases by a chemoresistive technique and reported the response/recovery time in order of seconds. The highest selectivity for acetone was found to be 71.32% among other test gases along with the lowest limit of detection (LOD) of 2 ppm acetone at RT with a response of 1.08, which is calculated as the ratio between the change in the electrical resistance of the sensor in the air to the test gases. In 2017, a porous coral ZnO was studied and reported by Zhu et al. for the detection of formaldehyde, methylbenzene, methanol, and acetone with a concentration of 600 ppm at 360 °C [74]. With this special porous structure of ZnO, the fabricated gas sensor revealed a substantial gas response with a fast response and recovery speed for all four different gases. Recently, in 2022, Swargiary et al. also developed ZnO nanorods coated over Single-mode–Mutlimode–Single-mode (SMS) fiber to detect isopropanol (IPA) vapor at various concentrations as a biomarker for diabetes patients [78]. The fabricated sensors exhibited a high sensitivity of 0.053 nm/% for IPA vapor detection at RT. Figure 5 shows the morphology of ZnO nanorods coated on the SMS for VOC sensing.

**Figure 6:**
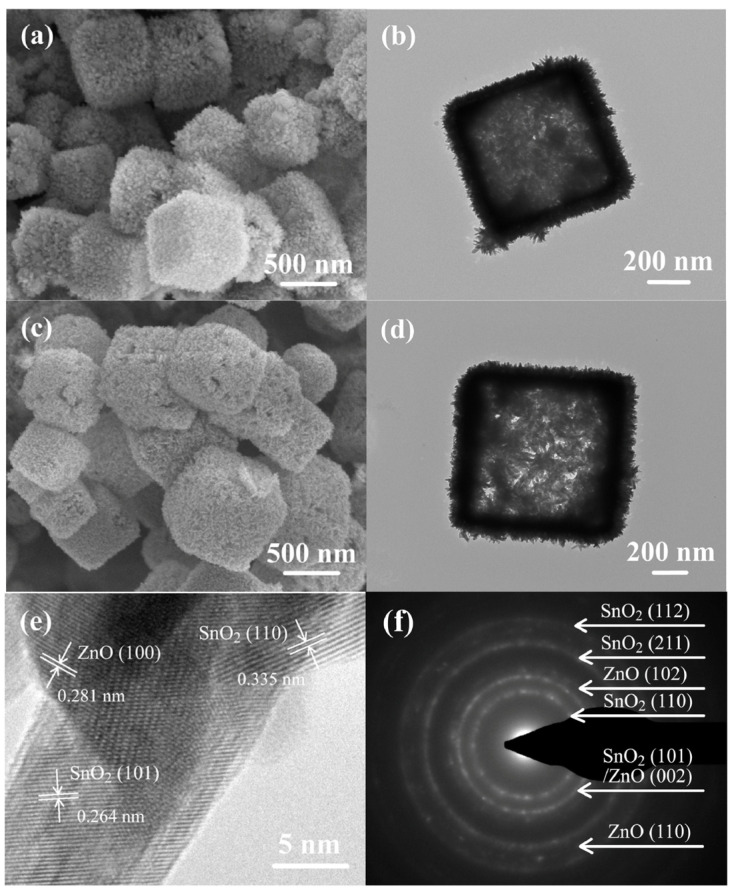
(**a**) SEM and (**b**) transmission electron microscopy (TEM) images of hollow SnO_2_/ZnS cubes. (**c**) SEM, (**d**) TEM, (**e**) high-resolution TEM images, and selected area diffraction (SAED) pattern of hollow SnO_2_/ZnO cubes (f). diffraction rings in the SAED pattern for different crystalline planes of SnO_2_ and ZnO (Reprinted/adapted from [88]).

Although the different ZnO nanostructures coated sensors have been reported to show an excellent response to the different VOCs, a high selectivity and sensitivity sensor to effectively detect multiple vapors in a mixed volatile environment is still challenging. For this reason, it can be considered as a drawback for using ZnO nanostructure alone as a sensing material for VOCs [85]. Some researchers reported that tuning ZnO nanostructures by doping, composite structures and/or using surface modification with different noble metals gives rise to the improvement of VOCs selectivity. In 2017, Ma et al. reported the Ag-doped ZnO/SnO_2_ sensor with hollow nanofibers in structures with a rapid response time of 5 s, with an excellent selective detection to 1 ppm ethanol at 200 °C. According to the report, a noble metal on the surface of ZnO is considered to be a site for adsorbate, catalysts, and surface reaction promoters and can improve the thermal stability of the nanostructure. The main advantages of this structure are its porosity and surface roughness morphology which makes it a promising candidate for efficient ethanol gas detection in the environment [86]. In the same year, Wang et al. exploited ZnO doped with Mn (MZO) to selectively detect 20 ppm acetone at 340 °C and CdO-activated MZO to selectively detect 20 ppm ethanol at 240 °C [85]. These gas sensors were also used as a sensor array to differentiate the acetone and ethanol mixtures at different ratios displaying the potential of a high-performance gas sensor. Furthermore, high selectivity and sensitivity with very stable response/recovery characteristics towards methanol among other VOCs were also reported by Mandal et al. with a rose-like ZnO microcube/MoO_3_ micrograss-based composite structure [87]. The most recent article reported in 2022 by Dong et al. prepared a novel composite of SnO_2_/ZnO hollow cubes that were synthesized by using a self-template hydrothermal method followed by a calcination process [88]. They explored the formation mechanism of the composite structure and its morphology, which is shown in Figure 6.

ZnO nanostructure formation was found to be on the thin wall of hollow cubes. SnO_2_ nanorods were found to adhere to those thin walls. With this structure, the VOC vapor sensing was carried out for formaldehyde, acetone, isopropanol, ethanol, n-butanol, methanol, benzene, and another three reductive gases (ammonia, hydrogen, and carbon monoxide) at 220 °C. Among all the VOC vapors, the hollow SnO_2_/ZnO cube sensor displayed excellent sensitivity, selectivity, and repeatability towards 100 ppm formaldehyde with stability for a long period of time and the highest response of 148 is achieved. This response was calculated from the ratio between the baseline resistance and the balanced resistance of the composite structure in the air and the test gases.

The selectivity of ZnO-based VOC sensors can be further improved by working on the features of the sensitive material as well as the operating parameters of the sensor. In addition, various limitations, and challenges, such as ultralow concentration detection, humidity interference from the individuals, and variability in VOC profiles of the individuals from the time of food intakes, have to be critically considered. Therefore, by changing the property of ZnO nanostructure morphologies with the doping and/or composite materials, a high selectivity sensor in a mixed gaseous environment can be accomplished with an effort to overcome the associated limitations. This can serve as the future development of multifunctional, compatible, and robust ZnO-based VOC sensors.

### 5.2. Nickel Oxide (NiO)

Nickel oxide (NiO) is an intrinsic semiconductor and is widely used for VOC sensing due to its unique characteristics such as wide bad gap, stability at high temperatures, transparency, and excellent chemical stability. The accomplishment of NiO depends on its morphology, dimension, and phase state such as porosity and dimensions.

**Figure 7:**
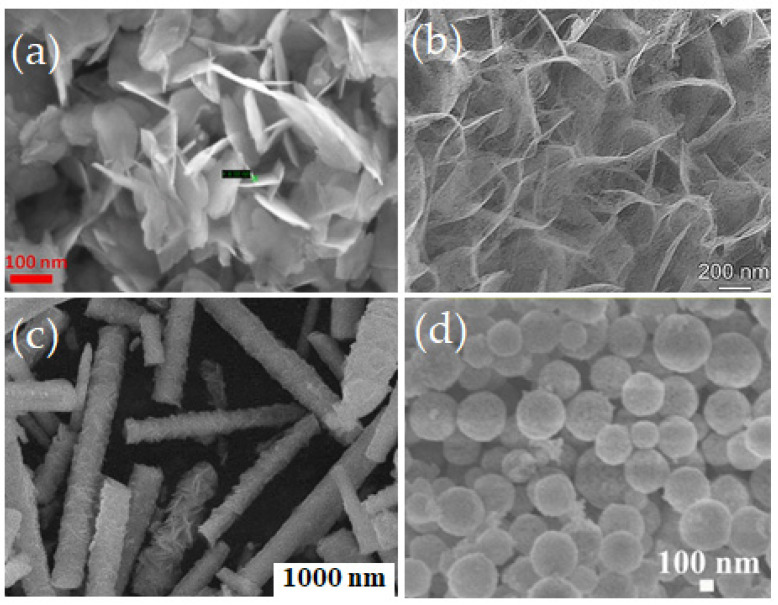
Field emission scanning electron microscopy (FESM) image of (**a**) NiO nanosheets [90] (**b**) NiO-75 (75% ethanol to distilled water ratio) thin nanosheets with porous structure ([89] (**c**) mesoporous NiO [91], and (**d**) 2.64% Sn-doped NiO [92].

In 2020, Nakate et al. studied the performance of the gas response of NiO nanosheets sensor via a hydrothermal chemical route which was prepared by mixing nickel nitrate solution and sodium hydroxide solution in de-ionized water [89]. The morphology of the NiO nanosheet is shown in Figure 7a. Ultra-thin nanosheet morphology of NiO can give a high surface area and active sites that play an important role in the high adsorption of gas to improve the efficiency of the sensor. The highest gas response reported in this work at 250 °C was 191% for 150 ppm hydrogen concentration with a response time of 150 s. In 2022, Li et al. developed ultrathin porous NiO nanosheets for acetone gas sensing by using the facile solvothermal method. They developed various NiO nanosheets on interdigitated electrodes by controlling the adjustment of ethanol to distilled water ratios, i.e., 0:8, 1:7, 1:3, 1:1, 3:1, and 8:0, which was referred to as NiO-0, NiO-12.5, NiO-25, NiO-50, NiO-75, and NiO-100, respectively. Among them, NiO-75 (75% ethanol solution-based NiO gas sensor) exhibited a high selectivity towards acetone at 225 °C, with a low LOD of 0.8 ppb along with a fast response and good stability due to its porous structure, large surface area and a crystal facet compared to the other NiO nanosheets as shown in Figure 7b [90]. The advantages of this method are low cost and easy to control. The obtained NiO nanosheet gas sensor (NiO-75) can be considered a suitable candidate for commercial acetone gas detection.

Meilin and Zhen developed mesoporous NiO for an ammonia sensor via the sol-gel method in 2019 [91]. The synthesized mesoporous NiO has a uniform rod structure with a pore size of 4 nm as shown in Figure 7c. The study involved stability which showed a long-term constant response from 0.2 ppm to 0.4 ppm ammonia concentration in 21 days at RT. In 2022, Yu et al. also reported a new development of the mesoporous oxide semiconductor gas sensor by introducing bio-metallic MOF-derived core-shell mesoporous Sn-doped NiO sensitivity materials via hydrothermal and ion exchange method, as shown in Figure 7d [92]. The sensor exhibited a high sensitivity of 100 ppm concentration of xylene at 250 °C with a low LOD of 63 ppb due to its high pore interconnectivity that increases gas diffusion on the surface.

In 2021, Ayyala et al. developed a chemoresistive NiO-based sensor for the detection of seven different common VOCs that include acetone, ethanol, toluene, hexane, methanol, 2-propanol, and isobutylene vapors between 5 ppm and 25 ppm at 350 °C and 40% relative humidity (RH) [93]. They deposited NiO using a spin-coating technique on the sensor with two different film thicknesses of 5 μm and 10 μm. The morphology of NiO film showed to be microporous structure and more compact due to the interconnections between the structure itself. The NiO sensor with two different thicknesses was reported to have a fast response and recovery time for all VOCs which were less than 80 s and 120 s, respectively. The highest response of 1.5 was also achieved and calculated from the ratio between the electrical resistance of air and the tested VOC, i.e., 5 ppm ethanol at 350 °C for both sensors. Recently, John et.al. investigated the crystalline NiO and manganese (Mn)-doped NiO nanostrcutures prepared by the co-precipitation method for the detection of VOCs including xylene, toluene, n-butyl, alcohol, 2-methoxyethanol, methanol, ethanol, acetone, ammonia, and formaldehyde [94]. These studies included the analysis of structural, elemental, morphological, and optical properties of the prepared gas sensor. The results showed that the Mn-doped NiO sensor exhibited high sensitivity, selectivity, and a rapid response time of 5 s, at 100 ppm concentration of formaldehyde along with a low LOD of 1 ppm at RT. This reported work also offered one of the cost-effective and facile methods for synthesizing NiO and Mn-doped NiO porous nanostuctures.

NiO has been studied to improve the sensitivity, selectivity, and stability of the VOC sensor by utilizing the sol-gel, solvothermal, hydrothermal, spin coating and doping methods. The main sensing mechanism of the NiO-based VOC sensors is generally based on the adsorption, desorption, and oxidation processes. The pore size of NiO can be modified for VOC’s detection that depends on the size of the targeted VOC molecules. Generally, the highly responsive sensors mostly have mesoporous structures due to their good volatile gas adsorption properties. However, there were reports showing that NiO-based sensors has a slow response time and low limit of detecting different clinical VOC biomarkers in real-time from patients suffering from bladder cancer and cystic fibrosis [95,96]. These sensors had a limitation in accurately detecting VOC concentrations as there exists a continuous change in the metabolic process in the human body that may give rise to multiple VOCs at a time.

### 5.3. Molybdenum Oxides (MoO_3_)

Molybdenum oxide (MoO_3_) is an n-type semiconductor that can detect several gases, e.g., carbon monoxide [97], hydrogen [98], methanol [99], etc. The main sensing mechanism of MoO_3_-based VOC sensors is the adsorption and desorption of gas molecules in the sensing area. The gas adsorption of the MoO_3_ sensor depends on its morphology which can be categorized into three types of structures. Firstly, a structure with a high aspect ratio is a one-dimensional structure (1D) such as nanofibers, nanorods, and nanoribbons. They have a limited surface area, but their reaction sites are mostly exposed to the environment. Next, is a two-dimensional structure (2D), such as nanoplates and nanosheets, which provides a larger surface area that can improve the sensitivity of gas detection. Lastly, three-dimensional structures (3D), such as nanoarrays, nanoflowers, and nanospheres, are assembled from low-dimensional structures providing the largest surface area and have more reaction sites for gas adsorption. A larger dimensional structure contains more gas diffusion channels, allowing gas molecules to pass into reaction sites. Therefore, it shows better gas absorption and sensing performance towards VOC molecules.

**Figure 8:**
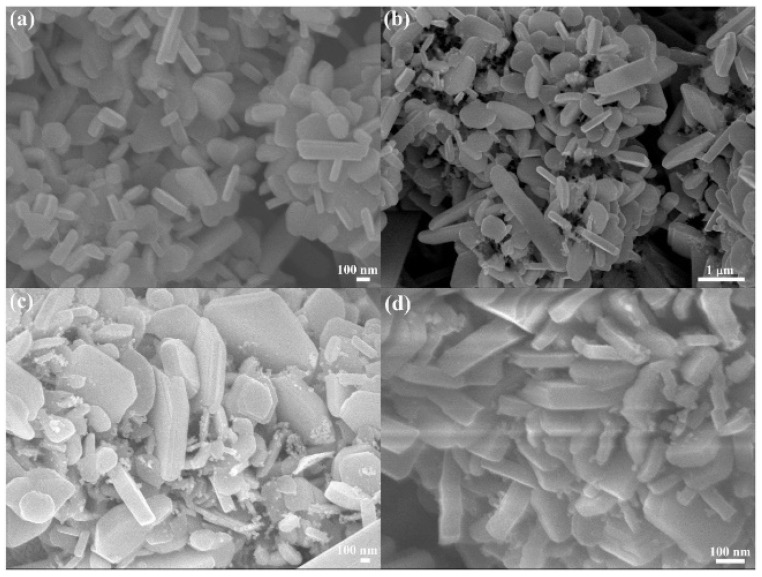
SEM image of Ni-doped α- MoO_3_ (**a**) 0 mol%, (**b**) 2.5 mol%, (**c**) 5 mol%, (**d**) 10 mol%. (Reprinted/adapted from [105]).

The MoO_3_-based sensor has a limitation in terms of selectivity. Therefore, the modification of the MoO_3_ surface to increase the specifically targeted gas adsorption ability is required. The MoO_3_ surface can be modified by doping with transition metals such as Cr, W, Ag, Au, Fe, Zn, Ni, or other nanomaterials to reduce the activation energy of chemosorption reaction. Furthermore, the composition of various materials to obtain hybrid structures, i.e., a mixed structure of two metals such as MoO_3_ and Au [100], α-MnO_2_, and h-MoO_3_ [101] affects the properties of sensing material in terms of its grain boundary barrier, energy band, carrier concentration, and depletion layer resulting in the sensing performance improvement [102]. In 2016, S. Yang, et al. developed the MoO_3_ nanobelt sensor synthesized from the hydrothermal method to detect trimethylamine (TMA) [103]. The sensing response was calculated from the ratio between the electrical resistance of the sensor in air and in testing gas. The sensor showed a response value of about 6, 36, and 582 towards 1 ppm, 5 ppm, and 50 ppm of TMA gas at 240 °C, respectively, with a response time of 15 s. In addition, this sensor showed a higher selectivity to TMA when tested with TMA, ethanol, methanol, acetone, ammonia, and toluene. It was also reported that not only does the surface morphology of MoO_3_ help to improve the sensing performance but the doping of MoO_3_ with noble material also plays a significant role in the improvement of sensing performance [104]. In 2017, Shen et al. reported the doping of Ni to α-MoO_3_ nanolamella for formaldehyde detection by using the solvothermal method [105]. The α-MoO_3_ was doped with various doping concentrations of 2.5 mol%, 5 mol%, and 10 mol%. The morphology of α-MoO_3_ doped with different concentrations of Ni is shown in Figure 8. They all have the same lamellar shape with a lateral length of 220 nm to 500 nm. With a 100 ppm concentration of formaldehyde at 255 °C, the 5 mol% doping concentration showed the best response with a response time of 37 s and a recovery time of 2 s.

**Figure 9:**
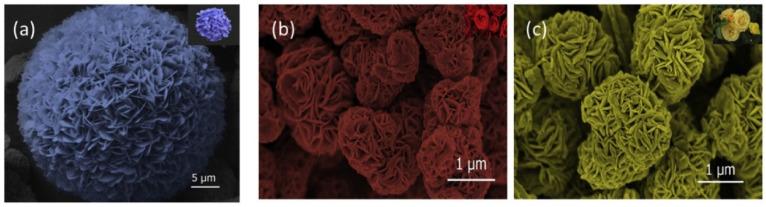
SEM image of MoO_3_ samples (**a**) sphere-like, (**b**) rose-like, (**c**) plate flower. (Reprinted/adapted from [108]).

In 2018, Wang et al. developed the multi-component structure of Au/α- MoO_3_ nanobelts for ethanol detection by applying gold nanoparticles onto α-MoO_3_ nanobelts using the hydrothermal method [106]. The rod structured morphology was observed to be homogeneous and uniform with an average rod length of 5 µm to 10 µm along with a 4.3 nm diameter of Au nanoparticle. With the exposure to 200 ppm concentration of ethanol, the Au/α- MoO_3_-coated sensor exhibited a rapid response with a recovery time of 4.3 s compared to a conventional α- MoO_3_ at 200 °C. The reported material has been investigated over ethanol, acetone, xylene, toluene, ammonia, and formaldehyde and observed to be highly selective for ethanol. In 2019 Shen et al. synthesized porous α- MoO_3_ nanosheets to detect TMA with a faster response and recovery time of 12 s and 200 s, respectively [107]. The highest response to TMA was achieved when the sensor was investigated with various VOCs such as acetone, formaldehyde, ethanol, ammonia, TMA, and dimethylamine. The sensor showed a response of 198 ppm to 50 ppm TMA concentration with a LOD of 20 ppb. In 2019, a flower-like MoO_3_ structure was assembled from nanosheets for ethanol detection by Ji et al. [108]. There were three MoO_3_ flower-like structures synthesized by the hydrothermal method including (i). sphere-like, (ii). rose-like, and (iii). plate flower as shown in Figure 9. The sensing performance of the materials was investigated at 50 °C to 350 °C under the controlled concentration of ethanol of 300 ppm. The obtained results showed that the rose-like nano flower provided the highest response of 37.1 compared to the sphere-like nanoflowers and plate flowers which were 30.9 and 27.3, respectively.

It can be observed that several MOs have been exploited on a large scale to develop a real-time VOC sensor. MoO_3_-based VOC sensors can selectively detect specific gases due to their different morphologies. To increase the sensitivity and selectivity of the MoO_3_-based sensor, a doping material with a transition metal element is recommended. This technique can increase the sensitivity by reducing the activation energy of the chemisorption reaction, leading to more interaction between reaction sites and targeted VOC. Moreover, adding metal components to obtain the hybrid component structure can also be considered for improving the response and recovery times.

### 5.4. Titanium Dioxide (TiO_2_)

Titanium dioxide (TiO_2_) is a high-resistance n-type semi-conductor with a band gap of 3 eV. It gains huge attention in various applications including solar cells [109], photocatalysis [110], chemical sensing [111], and gas sensing [112]. The major advantage of TiO_2_ is its chemical stability, catalytic properties, and easy modification of its optical, transport and structural properties which plays a significant role in gas-sensing applications [113]. Furthermore, TiO_2_ represents three crystal structures in nature which are (i) anatase, (ii) brookite, and (iii) rutile. The rutile structure is considered as the most stable, while the brookite and anatase structures are metastable and can be irreversibly converted to the rutile structure by annealing at 600 °C to 800 °C [114]. Anatase is widely used in gas-sensing applications due to its prominent gas reaction capacity and high oxygen vacancies [115]. However, the pristine TiO_2_ gas sensors still have limitations that include low selectivity, high operating temperatures, unstable repeatability, and stability when it is exposed to oxidizing gases. In past years, several TiO_2_ nanomaterials with new composites and structures have been reported and used in VOC monitoring [115]. The morphology geometry of TiO_2_ nanostructures can be synthesized in zero-, one-, two-, or even three-dimensional nanostructures [116]. The TiO_2_-based VOC sensors are mainly based on the adsorption phenomenon due to the presence of various active sites suitable for VOCs [5]. In this section, a detailed discussion has been provided on the various types of TiO_2_ structures and their role in the enhancement of the sensing performance of VOC gases. The TiO_2_ nanostructures are considered a potential sensing material for ultrasensitive and miniaturized vapor sensors due to their large specific surface area which provides various active sites for the adsorption of gases.

**Figure 10:**
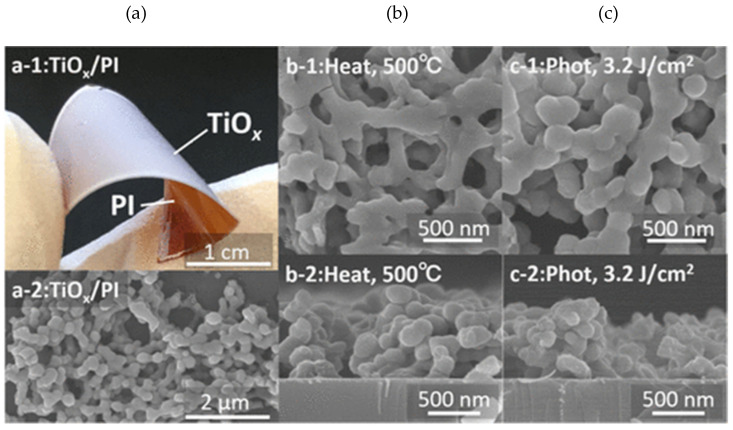
(**a**) Photograph and top view of the SEM images of TiOx nanostructure thin film on PI substrate after photosintering at 3.2 J/cm once on the PI substrate. Top view/cross-sectional view of the SEM images of TiO_2_ nanostructures on the rigid SiO_2_ glass substrate after (**b**) heat sintering at 500 °C and (**c**) photosintering at 3.2 J/cm one time. (Reprinted/adapted from [119]).

The 0D TiO_2_ nanostructures include nanospheres, quantum dots, and nanoparticles, which can be synthesized using various techniques such as the sol-gel method [117], hydrothermal [118], metal-organic decomposition (MOD) [119], and laser ablation in liquid [120]. In 2015, Li et al. reported hollow TiO_2_ microsphere-coated chemoresistive gas sensors for the selective detection of formaldehyde [121]. The sensor exhibited a high sensitivity and excellent selectivity to sub-ppm level of formaldehyde at RT along with an average response time of 40 s. The response time of the TiO_2_-based sensor was improved by other nanostructures [115]. In 2018, Navale et al. synthesized anatase TiO_2_ nanoparticle-based chemoresistive sensor for the real-time detection of acetone at ppb level at 270 °C [118]. One of the key features of the fabricated sensor was the rapid response time of 10 s and low LOD of 500 ppb of acetone. The sensor exhibited high selectivity towards acetone in the presence of ethanol, methanol, ammonia, nitrogen dioxide, and cyclohexanol. The high selectivity for acetone is attributed to the higher adsorption activities on the TiO_2_ surfaces. In 2020, Tohru et al. reported sphere-like anatase TiO_2_ nanostructures using the MOD technique, which is a process combining the coating and heat sintering or photosintering for VOC sensing, as shown in Figure 10 [119]. In this study, a thin film of TiO_2_ nanocrystalline structure was synthesized successfully. The fabricated sensor was able to detect 1-propanol with the highest response along with ethanol and methanol at concentrations varying from 50 ppm to 350 ppm. The sensor exhibited an excellent sensitivity of 40% to 60% with rapid response time of 1 s at 350 °C. The improved performance was achieved due to its large surface area and highly crystalline structure.

In 2021, Gakhar et al. synthesized fullerene-C_60_-encapsulated TiO_2_ nanoparticles for the selective and sensitive detection of formaldehyde [122]. The sensing material was synthesized via a chemical route using hydrated fullerene-C_60_ and sol-gel-derived undoped TiO_2_ nanoparticles. The average diameter of the C_60_-encapsulated TiO_2_ nanoparticles was observed to be 150 nm. The reported sensor exhibited a double response in the magnitude of 117% with high selectivity and a fast response time of 12 s for various concentrations of formaldehyde from 1 ppm to 1000 ppm. The efficient sensing performance towards the formaldehyde was achieved in C_60_-encapsulated-TiO_2_ nanoparticles due to (i) the extremely reactive surface provided by the oxygen-functionalized C_60_ and (ii) an electronic exchangeability between formaldehyde and the TiO_2_ nanoparticles via C_60_ layers.

**Figure 11:**
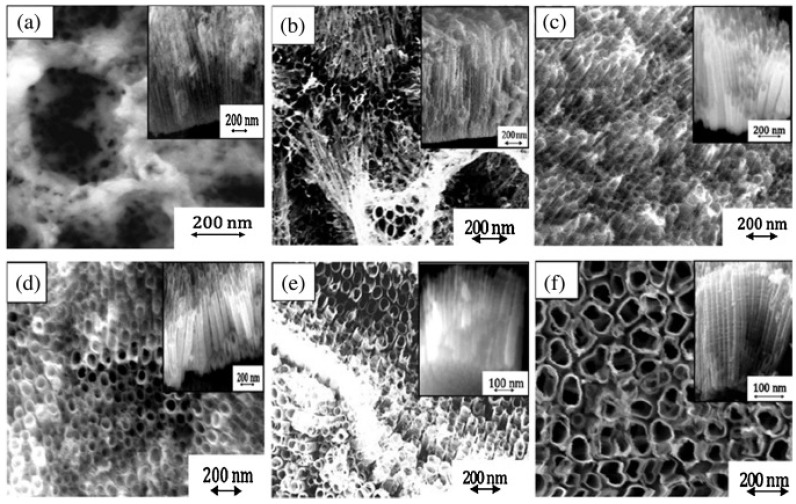
FESEM images of six anodically grown TiO_2_ samples (**a**,**b**) shows TiO_2_ nanotubes buried under fibrous coverage, (**c**) fibrous coverage is almost absent (**d**) nanotubular structures appeared (**e**) nanotubes were grown upward (**f**) free-standing ordered TiO_2_ nanotubes. (Reprinted/adapted from [119,135]).

The 1D TiO_2_ can be considered an ideal nanostructure for VOC detection due to its inherent properties of a strong adsorption capacity, large specific surface area, superior electron transport rate, and availability of the porous sites for faster diffusion of VOC molecules [123,124]. 1D TiO_2_ nanostructure includes nanorods, nanotubes, nanobelts, nanowires, and nanofibers. Several methods have been reported for the synthesis of 1D TiO_2_ including hydrothermal [125], electrochemical anodization [126], electrospinning [127], template-assisted synthesis [128], and matrix-assisted pulsed laser evaporation [129]. Among all these techniques, vertical TiO_2_ nanotube arrays synthesized by using electrochemical anodization contain more active sites for effective gas diffusion. The pristine 1D TiO_2_-based gas sensors have been successfully utilized to detect various VOCs, e.g., acetone [130], isopropanol [131], ethanol [132], formaldehyde [133], isopropanol [134], etc. In 2015, Dutta et al. performed electrochemically synthesis of TiO_2_ nanotubes for the real-time detection of benzene, toluene, and xylene (BTX) with enhanced sensitivity at a low temperature of 50 °C, as shown in Figure 11 [135]. Among these three VOC targets, benzene was observed to show the highest response magnitude followed by toluene and xylene at all concentrations varying from 20 ppm to 400 ppm.

In 2019, Bindra et al. fabricated a sandwich-structured sensor using Au/TiO_2_ nanotubes/Ti for the selective detection of four different VOCs including ethanol, methanol, acetone, and 2-propanol at RT [136]. The TiO_2_ nanotube array was grown via the electrochemical anodization technique which exhibited a maximum capacitive response towards the methanol compared to the other VOC test gases. However, it cannot completely recover its response for all other test gases. Researchers have also synthesized single-, double-, and triple-layered TiO_2_ nanotube arrays by using a voltage pulse-assisted anodization method [137]. In 2020, Zhao et al. synthesized a double-layered TiO_2_ nanorod array on fluorine-doped tin dioxide for the detection of low-concentration ammonia at RT [138]. The obtained results showed that the TiO_2_ nanorods have a growth time of nearly 6 h with a high gas-sensing response of 102% for 100 ppm to ammonia at RT. The most recent article reported by Cai and Park, in 2022, showed a synthesis of cobalt (II,III) oxide (Co_3_O_4_)-decorated porous TiO_2_ nanofibers by using the facile electrospinning technique to detect acetone [139]. The Co_3_O_4_ nanoparticle of diameter 30–50 nm was decorated on TiO_2_ nanofibers via the solvothermal process. The fabricated sensor exhibited a good response for acetone (71.88) compared to the other VOCs including ethanol (7.58), toluene (2.55), benzene (2.01), and p-xylene (3.01). In addition, the sensor exhibited an average response and recovery time of 122 s and 351 s, respectively, for 100 ppm acetone gas at 250 °C.

**Figure 12:**
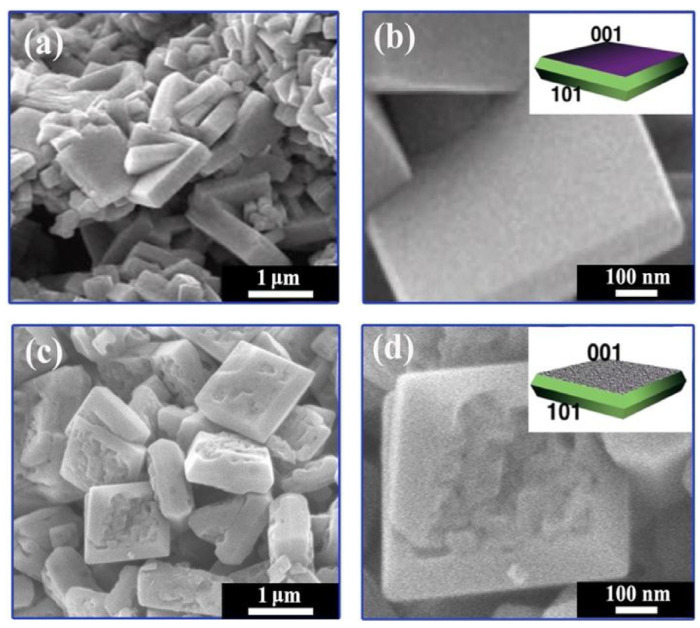
(**a**,**c**) The SEM images of C-TiO_2_ and D-TiO_2_, respectively; (**b**,**d**) the corresponding enlarged SEM images. The insets in (**b**,**d**) are the schematic illustration of different crystalline planes. (Reprinted/adapted from [150]).

The 2D TiO_2_ exhibited several versatile features including high specific area, flexibility, quantum Hall effect, and superior mechanical strength [140]. The 2D TiO_2_ nanostructure includes thin film, nanoplates and nanosheets. The 2D TiO_2_ subunits synthesized by using crystal facet engineering can significantly improve the selectivity and surface reactivity [141]. Several strategies have been employed to synthesize the 2D TiO_2_ nanostructures such as the chemical decomposition method [142], the one-step annealing process [143], reactive evaporations [144], and the hydrothermal method [145]. Among all of these techniques, the hydrothermal approach is the most common technique for the synthesis of 2D TiO_2_ nanostructures. In the past decades, the pristine 2D TiO_2_ nanostructures have been employed for a wide range of VOCs including isopropanol [5], methanol [146], acetone [147], hydrogen sulfide [148], and ammonia [149]. In 2019, Wang et al. synthesized TiO_2_ nanoplate-based micro-electro-mechanical system (MEMS) to detect acetone at RT, as shown in Figure 12 [150]. The author synthesized TiO_2_ nanoplates with defective and complete {001} crystal facets by varying the concentration ratio of hydrofluoric and hydrochloric acid during the synthesis. The results showed that the sensing performance of TiO_2_ nanoplates with complete {001} facets was 70% higher compared to the defective TiO_2_ nanoplates. The poor sensing performance of the defective TiO_2_ nanoplates is attributed to fewer adsorption sites on the surface. The TiO_2_ nanoplates with a volume ratio of hydrofluoric acid to hydrochloric acid of 2/50 exhibited good sensing responses of 21.8, 8.46, 4.32, 4.08, 6.64, and 2.73, for acetone, ethanol, toluene, methanol, isopropanol, and benzene, respectively, at 400 °C.

In 2020, Ge et al. synthesized hierarchical porous TiO_2_ hexagonal nanosheets obtained from the layered TiSe_2_ nanosheet templates in a one-step annealing process [143]. The fabricated sensor exhibited a high selectivity towards acetone of 200 ppm concentration along with ultrafast response times of 0.75 s at 400 °C. The transportation of electrons between TiO_2_ and acetone was achieved quickly due to the unique porous hierarchical structure of TiO_2_ nanosheets, crystal facet engineering and strong interface coupling. Recently, Tian et al. developed a MEMS sensor to study the influence of multi-layer of TiO_2_/SnO_2_ heterojunction on the detection of ethanol [151]. In the reported work, SnO_2_ was proposed due to its smooth surface and small particle size which advances the diffusion of ethanol. Although the sensor exhibited a slower response time of 33 s, it can effectively detect a lower concentration (50 ppm) of ethanol at 260 °C along with the sensor response value of 7.54. The sensing response was calculated from the ratio between the electrical resistance of the sensor in the air and the testing gas.

**Figure 13:**
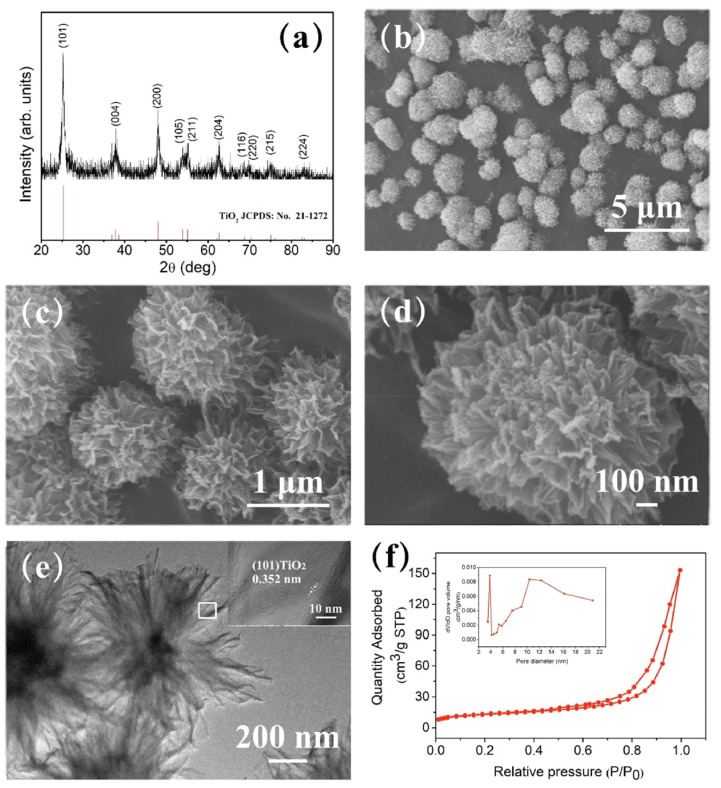
(**a**) XRD patterns, (**b**–**d**) FESEM images, (**e**) TEM image and HRTEM image (insert), and (**f**) BET results of TiO_2_ flower-like microstructures. (Reprinted/adapted from [156]).

Nowadays, 3D structured TiO_2_ nanoflower gains huge interest in VOC sensing. The TiO_2_ nanoflower structure comprises unique features allowing the diffusion and adsorption of VOC molecules onto its surface or penetrate into the interior part of these nanoflowers [152]. Moreover, some literature indicates that the TiO_2_ nanoflowers exhibit superior electronic properties compared to 0D, 1D, and 2D TiO_2_ nanostructures. This performance improvement comes from the structural defects, which act as active sites between the gas and TiO_2_ material [153]. The TiO_2_ nanoflowers can be synthesized by a facile hydrothermal method using titanium(III) chloride (TiCl_3_) [154], titanium tetrachloride (TiCl_4_) [155], Ti powders [156], titanium(iv) oxysulfate (TiOSO_4_) [157], Titanium(IV) isopropoxide (C1_2_H_28_O_4_Ti) [158], or Ti foil [159] as raw material. In 2016, Bhowmik et al. synthesized 3D TiO_2_ nanoflowers consisting of 2D nanosheets utilizing a low-temperature hydrothermal technique [160]. The fabricated sensor exhibited a high selectivity and rapid response time towards acetone at 60 °C. In this work, the authors considered the effect of the bond dissociative activation energy, vapor pressure of the individual VOCs, acetone selectivity, and the diffusivity of the gas species at the TiO_2_ nanoflowers surface in order to describe its performance at low operating temperature. They investigated the dual effect of the low bond dissociative activation energy and the higher VOC vapor pressure of the acetone molecules compared to the other VOCs (toluene, propanol, methanol, and butanone). The result showed a maximum response towards acetone. In 2020, Wang et al. synthesized 3D hierarchical TiO_2_ nanoflowers to detect ethanol varying from 10 ppm to 500 ppm at RT, as shown in Figure 13 [156]. This nanoflower-like TiO_2_ nanostructure is composed of thin nanosheets and was synthesized by the hydrothermal technique. The sensor exhibited good sensing performance in terms of good stability, high selectivity, rapid response/recovery times, and good reproducibility against ethanol vapor at RT. The nanoflower structure and the spacing intervals provide more adsorption sites for the oxygen species and the VOC gases, leading to exceptional gas-sensing performance of the material.

In summary, TiO_2_ can be categorized as 0D, 1D, 2D, and 3D nanostructures. Each nanostructure exhibited an excellent sensing performance towards VOCs including selectivity, sensitivity, and rapid response time. From the above discussions, it can be seen that the higher dimension structure of TiO_2_ showed rapid response and recovery time. The lower dimension structure provided a poor response, but it is easy to synthesize. Even though TiO_2_ alone can successfully detect several VOCs, it fails to show a selective response in the mixed gaseous environment. Therefore, the noble metal loading/composition technique has been utilized by several researchers to enhance the selectivity for target-specific detection.

### 5.5. Advantages and Limitations of MO-Based VOC Sensors

The performance of MO (such as ZnO, NiO, MoO_3_, and TiO_2_)-based VOC sensors can be improved by reducing the particle sizes to a nanoscale level in the sensing layer. Thus, a larger surface area and a higher active site can be achieved leading to excellent sensitivity. Changing the property of MO-based sensors by doping and/or adding composite materials may offer an option for improving the selectivity and LODs. However, there still exist numerous challenges and limitations on VOC profiling for clinical samples in a multivariate environment and conditions. The MO-based sensors should also be able to overcome the major limitations of the VOC profiling approach such as the extremely high costs of the laboratory instruments based on accurate VOC detection, and standardized sample collection for clinical trials. In addition, the detection of ultralow concentrations of associated VOCs from te different persons, the development of generic MO-based sensors for their durability, robustness, selectivity, reliability, energy efficiency, and standard calibration of these sensors upon malfunction still remain a challenge.

In Table 3 we have summarized the various kinds of metal oxide sensors and their sensing performances.

## 6. Carbon Based Materials and Composites

### 6.1. Graphene Oxide (GO)

The usage of GO is considered another potential candidate for non-invasive VOC vapor detection due to its unique and desirable properties such as being thermally stable, large surface area, and highly electrically conductive. These properties of GO have been exploited over the past years by several researchers to detect VOC vapor. The mechanism of graphene and its derivative sensors is based on the change in the resistance that is associated with the adsorption/desorption of the VOC molecules on the graphene surface which leads to a change in its properties. These VOC molecules can easily adhere to the large surface area of the graphene, changing the conductivity of the graphene sheet [186,187,188,189]. The change in conductivity can be used to measure the concentration of the VOC vapor in the environment with a high level of sensitivity due to the large amounts of charge carriers and electron mobility. However, due to the current availability of fabrication techniques and technologies, it is extremely difficult to fabricate pure graphene using economically viable techniques. Therefore, most applications that require the usage of graphene-based structures rely on GO, which can be mass-produced by using chemical exfoliation. On the other hand, the usage of GO to detect the VOC vapor have several limitations such as a reduction in the level of conductivity due to oxygen and other oxygenated groups inside the graphene matrix which reduces the sensitivity of the VOC vapor sensor. This allows the graphene structure to be more vulnerable to a humid environment [190]. To overcome this limitation, reduced graphene oxide (rGO) is often used instead of pure GO. rGO contains a lower level of oxygen and oxygenated groups in the graphene matrix, allowing it to function more like pure graphene and minimizing the negative effects of the oxygen and other oxygenated molecules. However, the usage of rGO alone is not efficient because of the other types of VOCs that are attached to the sensor, resulting in low selectivity. Therefore, it is usually more efficient to utilize rGO with another compound or material to make the rGO structure more selective to the target VOCs.

**Figure 14:**
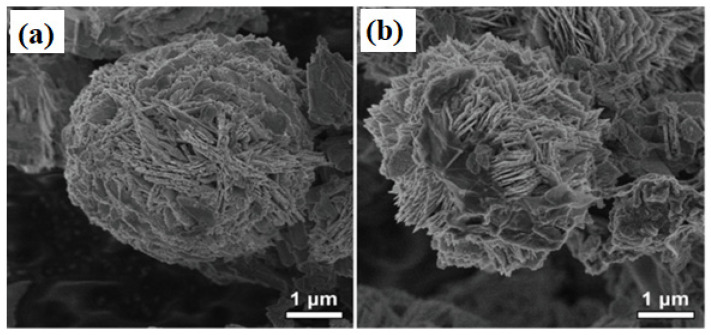
SEM image of (**a**) CuO hierarchical structure and (**b**) integration of rGO and the CuO hierarchical structure. (Reprinted/adapted from [192]).

One example of the integration of rGO can be seen in the works of Zito et al. reported in 2018, where a combination of rGO sheets and NiO composite in the hierarchical flower-like structure was used to detect methanol [191]. Integrating rGO increases the number of holes on the surface of NiO due to the transfer of the charged carriers to balance the difference in the Fermi levels of rGO and NiO. Oxygen is oxidized in the presence of methanol, allowing the electron to recombine with the hole. This increases the resistance, which is used to measure the concentration of methanol. Thus, the sensitivity and response of the sensor can be improved. In 2019, Modenes et al. worked on rGO combined with copper (II) oxide (CuO) hierarchical structure to create a VOC vapor sensor for ethanol detection [192] as shown in Figure 14. Figure 14a shows a FE-SEM image of the CuO hierarchical structure that was used as the basis of the VOC vapor sensor, while Figure 14b is the integration of CuO and rGO, where rGO is composed of 2% of the weight of the entire solution. Even though CuO has previously been used to detect gaseous particles such as VOC vapor, using it alone gives a low level of sensitivity. Therefore, integrating rGO with CuO allows the VOC sensor to take advantage of both materials to detect ethanol. With the presence of ethanol particles, the ionized oxygen adhered onto the surface will be oxidized, resulting in electron-hole pair recombination. This reduces the overall level of CuO conductivity. When oxygen atoms adhered onto the surface of CuO, oxygen is ionized into a negatively charged ion, which leaves a hole in the CuO matrix as CuO is also a p-type semiconductor. These holes accumulate on the surface of CuO, thus allowing the surface to be more conductive and sensitive to ethanol.

In the same year, Pargoletti et al. fabricated a novel composite material based on GO sheets that were decorated with tin dioxide (SnO_2_) nanoparticles by controlling chemical growth. They reported the composite materials, SnO_2_-GO (4:1), SnO_2_-GO (8:1), and SnO_2_-GO (16:1), and pristine SnO_2_ material for the detection of ethanol at low concentrations. Among them, the hybrid material SnO_2_-GO with a ratio of 16:1 was found to be superior for ethanol sensing at 1 ppm concentration both at RT (exploiting the UV light) and 150 °C with response and recovery times of 310 s/320 s and 70 s/75 s, respectively [193]. In 2020, Wu et al. reported a NO_2_ vapor sensor using a combination of rGO and SnS_2_ [194]. rGO was synthesized into a 3D porous structure to increase the surface area and the density of sites in such a way that NO_2_ particles can adhere to the surface. SnS_2_ is integrated into rGO to increase the selectivity as it is an n-type semiconductor that has a special affinity allowing the adsorption of NO_2_ molecules due to the high electronegativity of a semiconductor. However, SnS_2_-based sensors are not usable at RT when used alone due to a high electrical resistance value. Therefore, integrating rGO with SnS_2_ to develop a NO_2_ sensor can allow rGO to benefit from SnS_2_ by achieving a high level of selectivity for NO_2_ gas, while SnS_2_ can utilize the superior level of conductivity from rGO to overcome its inherent resistance value and operate within RT.

The sensitivity of GO and rGO VOC vapor sensors can also be improved by integrating them with nanostructured materials. In 2021, Zhao et al. reported the use of rGO in conjunction with SnO_2_ nanorods to develop a formaldehyde sensor [195]. The sensing efficiency of SnO_2_ depends on the morphology. In this report, the SnO_2_ nanorods structure is the most suitable structure integrating with rGO. The sensor showed a 150-times better response to formaldehyde due to larger available sites from the nanorods structure. Another research on Ag nanoparticles modified with Fe_3_O_4_/rGO composite through hydrothermal method for the detection of acetone was done by Jia et al. in 2022 [196]. The composite structure Ag/Fe_3_O_4_/rGO showed an ultra-high response (R_a_/R_g_ = 35.81) at 50 ppm acetone concentration at 220 °C which was 2.5 times higher than pure Fe_3_O_4_/rGO composite structure. This is due to the chemical and electronic excitation effects of Ag nanoparticles which generate more oxygen species and active sites for excellent gas sensing performance. Moreover, introducing the noble metals along with the metal oxide and rGO offers excellent sensitivity, good response, and selectivity of the device for practical applications.

In 2019, Wang et al. developed a ternary nanocomposite NO_2_ sensor consisting of rGO, ZnO and SnO_2_ [197]. They reported that the NO_2_-sensing properties of the sensors on rGO improved massively from the formation of the heterostructures between the two metal oxides. With this nanocomposite, the electronic properties of the hybrids and the band structure change thereby promoting effective charge transfer among the interfaces of heterostructures which improves the gas-sensing performance. With this advantage of ternary structure-based sensors, a similar kind of work was also reported by Sen et al. in 2021 using the same materials for the detection of VOC vapor [198]. Therefore, these constructions of heterojunction have displayed the potential for enhanced VOC detection applications as well. ZnO is one of the building materials in a VOC sensor because it has a good level of response to gas molecules and has high stability in many operational environments. However, it must be integrated with other materials such as rGO to minimize a level of resistance. By adding ZnO to SnO_2_/rGO sensor, a p-n-n heterojunction is created allowing a larger surface area for the VOC vapor absorption. This can significantly increase the sensitivity and response of the sensor for multi-VOC vapor detection in mix gaseous environment.

Most rGO and GO-based VOC vapor sensors are usually fabricated to detect/target only one type of VOC vapor species which is the main limitation of this sensor. Therefore, it is highly desirable to develop a sensor that can be reliable, efficient, cost-effective, and possess a standard calibration for detecting multiple types of VOC vapor in terms of a clinical point of view in real-world applications. rGO is incorporated with other materials to take advantage of the desirable properties of each material to improve the VOCs sensing ability [10]. In addition, other types of rGO and GO-based sensors such as a ternary-based sensor also offer the ability to detect multiple VOCs.

### 6.2. Multiwall Carbon Nanotubes (MWCNTs)

Carbon is one of the most abundant elements on the planet. It is a promising material for nanoelectronics as it is able to form itself into various crystal structures, resulting in a different material property. Due to its superior properties from the four valence electrons, allows the mixing of s and p atomic orbitals to form several hybridization, i.e., sp^2^ and sp^3^ hybridization. The perfect hybridization provides a higher electrical conductivity, larger specific surface area and other improved electrochemical properties [199]. In addition, carbon can be formed into various nanostructures such as fullerene zero-dimensional structure, carbon nanotube one-dimensional structure, graphene two-dimensional structure, fullerite 3D structure, and carbon nanotube which have an extraordinary advantage in VOC sensing. The primary sensing mechanism of MWCNT-based VOC sensors is the adsorption of VOC molecules on the surface leading to a change in the output of the sensor.

A carbon nanotube (CNT) is a two-dimensional cylindrical structure with the thickness of one graphene arranged in a hexagonal lattice where each atom forms covalently with the sp^2^ hybridization that is even stronger than the diamond lattice formed in the sp^3^ hybridization [200]. CNTs have been widely reported with superior electrical, mechanical, and thermal properties that can be up to multiple times greater than typical steel or even some class-leading materials [201]. For this reason, there are many possibilities in several potential applications ranging from material enhancement, surface modification, biological applications, and electronic applications [202]. Many researchers have reported that the CNTs are able to transport electrons without any scattering phenomena due to their hollow shape in angstrom (*Å*) diameter. Therefore, the electrons can be quantized and confined along the circumferences and proceed only in the axial direction with a thousand times better mean free path compared to copper. Furthermore, the resistivity of the CNT can be as low as microohm per centimeter range [203]. The superior mechanical properties can be described as up to 63 GPa in tensile strength and 1 TPa in Young’s modulus which is greater than the 10-fold industrial-grade fibers [204]. The ideal CNTs have very high thermal conductivity, approximately 3000 W/m·K, which is eight times greater than copper and two- times greater than the copper diamond with sp^3^ hybridization [205]. In addition, Deng et al. reported in 2014 that the coefficient of thermal expansion of CNT can be as low as 1.9 × 10^−5^ K^−1^ at RT which hiders the change in shape allowing more possible applications [206].

**Figure 15:**
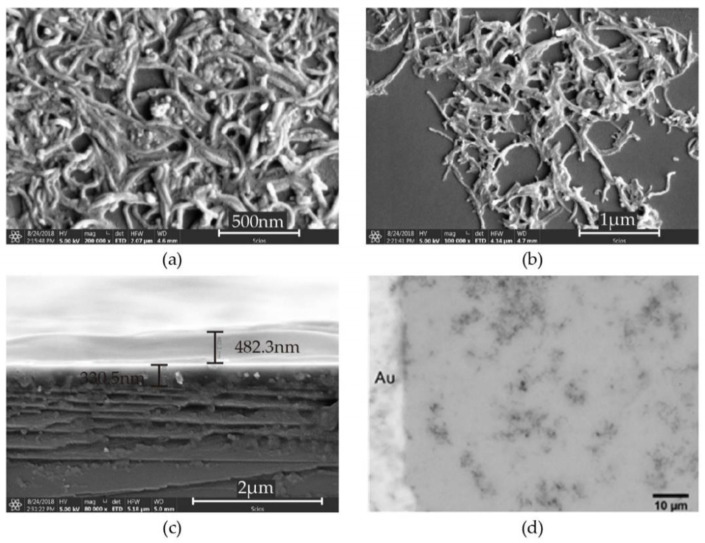
FESEM images of (**a**) MWCNTs on silicon dioxide; (**b**) PEG/MWCNTs on silicon dioxide; (**c**) the cross-section of MWCNTs and PEG composite layer. (**d**) Optical microscope images of MWCNTs and PEG composite layer. (Reprinted/adapted from [209]).

Carbon atoms can be arranged in a variety of nanostructures including CNTs which can be categorized into two major types, single-walled carbon nanotubes (SWCNTs) and MWCNTs. CNTs can also be categorized according to their chirality to be armchair, zigzag, and chiral, which represent their electronic properties to be metallic-like or semiconductor-like properties. The MWCNTs have a large surface area, high conductivity, and excellent flexibility in chemical stability compared to the SWCNTs [207]. Therefore, it is considered the preferred candidate for the VOC gas-sensing applications. In 2017, Bahoumina et al. reported a microwave flexible VOC sensor based on poly (3,4-ethylenedioxythiophene) polystyrene sulfonate–multi-wall carbon nanotubes (PEDOT:PSS-MWCNTs) [208]. The sensing material was deposited using inkjet printing technology. The sensing response of the device was estimated at 4 min and 10 min exposure to ethanol vapor at 0 ppm, 500 ppm, 1000 ppm, and 2000 ppm concentration. The maximum sensitivity of the device has been estimated at −642.9 Hz/ppm and 648.1 Hz/ppm at 4 min and 10 min exposure of ethanol, respectively. In 2018, Liu et al. developed a RT VOCs vapor sensor utilizing layer-by-layer MWCNTs/poly-ethylene glycol (PEG) composite, as shown in Figure 15 [209]. The sensing performance of the device was calculated by measuring the relative resistance change when the sensor was exposed to the VOC’s environment. The sensor exhibited a high sensitivity to acetone, isopropanol, ethanol, and isoprene along with a rapid response time of 110 ± 5 s and a lower LOD of 9 ppm at RT.

**Figure 16:**
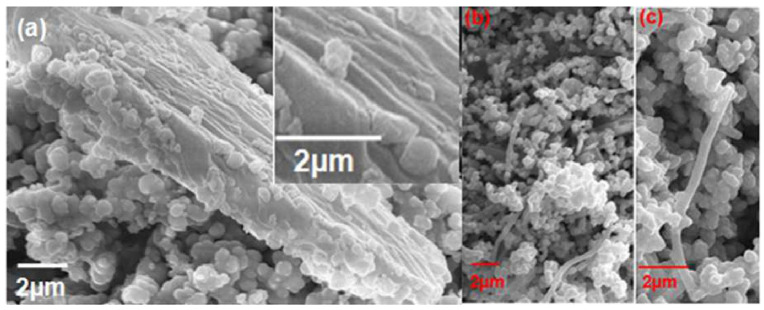
SEM images of two different samples (**a**) MoS_2_ only (**b**,**c**) MoS_2_ and MWCNTs hybrid. (Reprinted/adapted from [211]).

In 2019, Janfaza et al. reported a highly selective chemoresistive sensor to detect hexanal composite layers of molecularly imprinted polymer (MIP)/MWCNTs on RT [210]. The result exhibited that the electrical resistance was increased with the concentration of hexanal due to the interaction of the vapor with functional groups on MIP leading to the swelling of the MIP. The MIP swelling allows moving MWCNTs away from each other and changing the conducting path created by MWCNTs in the sensitive film, leading to a change in the resistance of the sensor. Therefore, a significant increase in the resistance of the sensor occurs when interacting with hexanal. The sensor showed a linear sensing response with a low LOD of 10 ppm for the concentration varying from 10 ppm to 200 ppm. In 2020, Singh et al. synthesized molybdenum disulfide (MoS_2_)/MWCNTs composite layer using hydrothermal route to detect ammonia ranging from 12 ppm to 325 ppm, at RT [211]. In this work, a comparative study has been performed on two configurations Ag/MoS_2_/Ag and Ag/MoS_2_/MWCNTs/Ag. The device exhibited n-type semiconducting behavior and is capable of detecting ammonia as low as 12 ppm. The SEM image of MoS_2_ and MoS_2_/MWCNTs is shown in Figure 16. In the case of the MoS_2_ configuration, the sensor exhibited a slow response time of 400 s which was later significantly improved by the MoS_2_/MWCNTs composite layer to be 65 s. It has also been discussed in the report that these significant differences appeared due to the different adsorption energy for different VOC molecules on the surface of MoS_2_ or MoS_2_/MWCNTs composite.

The sensing performance of MWCNTs was later improved in 2022 by Yan et al. utilizing polypyrrole nanocomposite-coated with MWCNTs [212]. Thermoplastic polyurethane (TPU) conductive nanocomposites were fabricated using TPU as a matrix and polypyrrole (PPy) coating MWCNTs as a conductive filler. The fabricated TPU/MWCNTs-g-PPy nanocomposite comprised certain gas-sensitive response selectivity, especially for ammonia gas compared to the other VOC molecules. The sensor exhibited improved response values in the range of 90% to 100% with a rapid response time of 40 s to 50 s.

MWCNT can be considered one of the promising candidates in the VOC sensing application due to its superior properties, e.g., electrical, mechanical, and thermal properties. However, the major limitation of the MWCNTs sensor is the deterioration of the structure during long exposure sessions of the VOCs. This can cause a false output measurement which can be very crucial in some applications. Nonetheless, these challenges can be minimized by integrating with composite materials such as PEG, MoS_2_, PPy, MIP, etc. These composite materials not only solve the problem of deterioration of material but also improve performance in terms of sensitivity and response time.

### 6.3. Activated Carbon (AC)

With the rapid progress of industrialization and constant development of urbanization, VOCs and their toxicity have been a serious concern not only in the environment but also for human health [213]. With the constant increase in VOC emission, various techniques to degrade the VOCs are being investigated and implemented. One of the techniques is the add-on control where the VOC is absorbed by absorption materials due to physical and chemical interaction in exhaust gas molecules [214]. This technique allows effective, efficient, and economic measurements [215].

**Figure 17:**
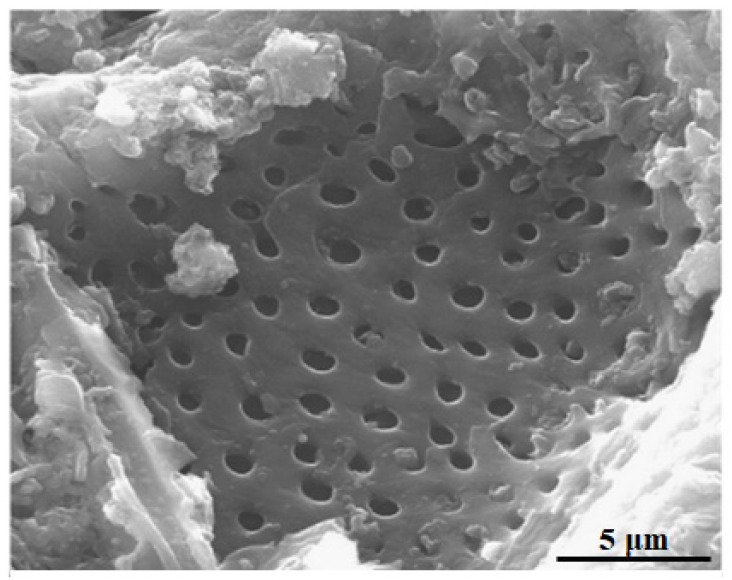
SEM image of palm shell-based AC prepared under optimum conditions. (Reprinted/adapted from [220]).

ACs are a type of absorption material that has been widely used due to their good physical and chemical properties with a large specific surface area, high chemical stability, high mechanical strength, and abundant functional groups along with alkali and acid resistance. Although ACs have these advantages, they also possess limitations due to their thermal stability and hydrophobic property. The ACs spontaneously ignite or collapse in a porous structure at high temperatures as it is a nonpolar absorbent. Therefore, it limits the adsorption towards hydrophilic VOCs [216,217,218]. ACs are generally produced in carbonization and activation process by carbon-rich materials such as coconut shells, wood, sawdust, lignin, palm shell, wood, petroleum pitch, and coal [219]. Figure 17 shows the SEM images of the precursor (raw palm shell AC) obtained under the optimum conditions developed by Arami et al. in 2012 [220].

There are several studies on ACs for the VOCs adsorption and its recovery such as alkane, alcohols, ethers, aldehydes, ketones, esters, aromatics, etc. In 2012, Li et al. investigated the influence of the ACs pore structure on the adsorption of VOCs [221]. They reported the fixed-bed thermostatic adsorption at certain conditions where the adsorption capacity of ACs to acetone, toluene, and 1,2-dichloroethane was conducted. The obtained results showed the selectivity of the adsorption of organic gases and the VOCs including toluene, acetone and 1, 2-dichloroethane adsorbed on the AC are in the different pore diameter range of (1.27 to 1.49) nm, (0.67 to 0.84) nm, and (1.39 to 1.75) nm. In 2019, Shen et al. prepared AC from rice husk (RH) and investigated the adsorption performance of toluene and phenol [222]. They reported the adsorption capacity for toluene (264 mg/g) to be higher than phenol (6.53 mg/g) in the gaseous phase. This is due to toluene being one of the strong volatile compounds that are greatly absorbed by RH char in ambient conditions. On the other hand, phenol is a semi-volatile organic compounds (SVOCs) that has a weaker volatility in the ambient, thus possessing a weaker adsorption capacity in the gas phase compared to the liquid phase. They also reported that AC can be reusable through thermal desorption. In 2020, Kang et al. proposed a gas concentrator for breath analysis of individual health to diagnose diseases by evaluating the adsorption and desorption performance from a commercial photoionization detector (PID) and a colorimetric tube detector [223].

The VOC investigated in this study was 1-ppm propionic acid. Its adsorption and desorption rates obtained were approximately 90% and 70%, respectively, making it an excellent gas concentrator which is benefited by low concentration for VOC detection. In the same year, Ma et al. studied the adsorption and adsorption mechanisms of toluene and chlorobenzene by two commercial ACs under medium-high-temperature (MHT) conditions [219]. The two ACs are coconut shell-based AC (CSAC) and wood-based AC (WAC) which were investigated in a fixed-bed reactor under medium-high temperature (MHT: 90 °C to 150 °C). The obtained results showed that the toluene and chlorobenzene adsorption characteristics at MHT conditions were different from those at low temperature adsorption. The adsorption values of toluene on CSAC and WAC at MHT conditions were 20.6 mg/g to 49.9 mg/g and 13.9 mg/g to 46.9 mg/g, while for chlorobenzene were 30.5 mg/g to 80.3 mg/g and 26 mg/g to 63.9 mg/g, respectively. The results provided the basic data and theoretical references for the development of adsorbent injection coupled with bag filtering technology to remove organic pollutants. From the above study, we can see that the mechanism of the AC-based VOC sensors is mainly reported in terms of adsorption rates, adsorption capacities, and their operation under the controlled temperature. However, the response and recovery times of the AC-based sensor are still limited. Moreover, limited studies can be found on the selective optimum adsorption capacity for VOC mixtures. Thus, tailoring the physical and chemical properties of AC, such as integrating with doping materials, can enhance the VOC adsorption and allow the detection in sub ppb/ppm level which is a key criterion for clinical VOC biomarkers detection. ACs have promising properties such as large surface area, variable surface chemical functionalization, and tunable micropore size and volume. Therefore, AC is considered to be a very good candidate for achieving high adsorption efficiency/capacity for the VOC detection ranging from dozen to several hundreds of mL/g.

### 6.4. Advantages and Limitations of Carbon-Based VOC Sensors

Nowadays, the development of the high-performance VOC gas sensor using carbon and carbon composite-based sensing materials offer great advancement and progress in terms of the structure design [224]. However, the developed VOC sensors using carbon-based materials have not yet succeeded in achieving and realizing their commercialization in environmental gas detection, diagnosis of non-invasive disease, and a portable health-monitoring device. The complexities of the sensing material require the fabrication of a high-performance VOC sensor. The field needs to pay a continuous effort for further improvement of the overall sensing properties of VOCs sensors employing carbon-based nanomaterials, including sensitivity, specificity, low LOD, RT operational, rapid response, fast recovery time, good reproducibility, long-term stability, and capability of intelligent readouts.

In Table 4 we have summarized various kinds of carbon-based VOC sensors and their sensing performances.

## 7. Polymers

A polymer is a substance or material consisting of very large molecules called macromolecules, made by linking up smaller repeating chemical units. In the past decades, polymers have gained huge interest in the field of the development of artificial sensors due to their special characteristics of swelling and shrinkage in the presence of gaseous molecules [243]. Rapid response time and better selectivity have been achieved by replacing classical sensing materials with polymers involving nanotechnology and exploiting either the extrinsic or intrinsic functions of polymers. Polymers play a crucial role in the detection of various chemical and biological fluids due to their swelling and shrinkage characteristics [244,245,246].

### 7.1. Poly (Dimethylsiloxane) (PDMS)

Poly (dimethylsiloxane) (PDMS) is a silicon-based elastomeric polymer which is formed by the cross-linked structure of the repeating units of -OSi (CH_3_)_2_- groups. PDMS has been considered an ideal material for the fabrication of integrated circuits and microfluidic channels [247,248]. Furthermore, it plays a crucial role in VOC sensing over the last decades due to its swelling property in the presence of VOC molecules [249]. The swelling and shrinkage of PDMS in presence of various VOC concentrations leads to a change in the output of sensing devices.

**Figure 18:**
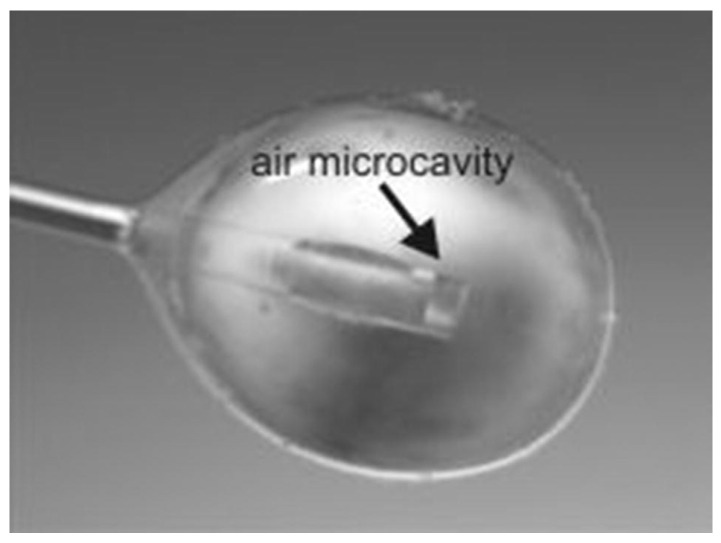
Microscope image of the fabricated microcavity inside PDMS. (Reprinted/adapted from [252]).

In 2012, Reddy et al. developed Fabry–Pérot interferometer (FPI) based optical vapor sensor array for micro-gas chromatography (μGC) applications [250]. The reported sensors were made by depositing a uniform thin layer of polymer on a silicon wafer. The polymer–silicon and air–polymer interfaces form the Fabry–Pérot (FP) cavity in which the resonance wavelengths varied with the VOC vapors allowing the rapid detection and quantification of VOCs. For the proof-of-concept, two polymers, namely PDMS and SU-8, were utilized independently and placed in a microfluidic channel. The sensor exhibited different sensitivities for the different vapors. It showed an excellent response to toluene and acetone for a concentration ranging from 0 ng to 20 ng and 0 ng to 150 ng, respectively. In 2016, Ning et al. reported a sensitive FPI and Sagnac Interferometer sensor coated with PDMS for the simultaneous detection of multiple VOC targets [251]. From the fabricated device, authors have successfully detected ethanol and 2-propanol by using second-order inverse matrix approach. The sensing head was fabricated using a single-mode hollow-core optical fiber coated with a 15 μm thick PDMS on the tip of fiber. The air between fiber and PDMS represented the FPI cavity (detail information on this interferometry technique is provided in our most recent review article [17]). The reported sensor exhibited a good sensitivity of 1.61 × 10^−3^ nm/ppm and 1.17 × 10^−3^ nm/ppm for 2-propanol and ethanol, respectively. Later in 2017, Kacik and Martincek utilized a similar approach to detect toluene using FPI formed by a micro air-cavity inside the PDMS matrix coated at the fiber-end facet [252], as shown in Figure 18. The interface between the fiber/air and air/PDMS served as two reflecting surfaces for reflected beams to form an interference pattern. The sensor exhibited an average sensitivity of 0.15 nm/g.m^−3^ to 1.4 nm/g.m^−3^ for the toluene concentration varying from 0.833 g·m^−3^ to 100 g·m^−3^.

**Figure 19:**
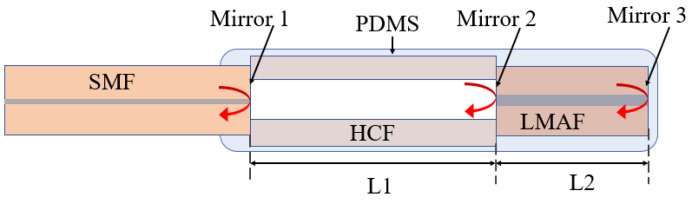
Schematic diagram of the fabricated FPI sensor coated with PDMS.

In 2019, Zhao et al. developed PDMS coated optical fiber sensor to form a FPI structure, as shown in Figure 19 [253]. The 1 mm long fabricated sensor was made from a small section of large mode area fiber (LMAF) and a segment of hollow-core fiber (HCF). The sensitivity of the device was determined by monitoring the interference spectral due to the Vernier effect. The fabricated sensor exhibited an excellent sensitivity of 20 pm/ppm for isopropanol varied in the range from 0 ppm to 500 ppm.

Recently, Sappati et al. reported a printed acoustic wave sensor with PDMS as a sensing layer for the detection of the low concentration of VOCs [254]. In this work, an acoustic flexural plate wave sensor was printed with silver ink on a thin and flexible lead zirconate titanate-PDMS composite layer. The prototype resonator showed a resonant frequency of 22.65 MHz with an attenuation of −1.552 dBm. The gravimetric mass sensitivity of the device was monitored by introducing PDMS layers between the input and the output interdigital transducers. The sensor exhibited a high sensitivity of 0.66 kHz/ppm and 160.63 kHz/ppm and a low LOD of 10.9 ppm and 0.03 ppm for acetic acid and toluene, respectively.

### 7.2. Conducting Polymers (CPs)

Monitoring VOCs using conducting polymers (CPs) is at the core of attention for the fabrication of next-generation sensor applications. The CPs are recognized as a class of organic materials with unique optical and electrical features similar to those of metal and inorganic semiconductors. The major advantages of these polymers are their processability [255]. CPs can be synthesized using versatile, simple, and cost-effective approaches which makes them potential candidates in sensing applications. CPs are composed of functional groups which have pseudo capacitance features and hence exhibit conductivity as the material itself such as polyacetylene, polypyrrole, polyaniline, polythiophene, poly(phenylenevinylene), poly(para-phenylene), and polyfuran. In the next subsections, we briefly discussed two major CPs, polypyrrole and polythiophene, and their roles in VOC sensing. CP is an important sensing material based on swelling and shrinkage phenomena to monitor the changes in VOC concentrations.

#### 7.2.1. Polypyrrole (PPy)

In recent years, PPy has been exploited in VOC sensing due to its salient features of high selectivity and sensitivity towards the detection of inorganic and organic gases [256]. PPy, is considered one of the emerging sensing materials with a wide range of applications in the field of electronic, optical, and electrochromic devices and sensors. It becomes a promising alternative in sensor development due to its stability in a different gaseous environment, ease of deposition from aqueous and non-aqueous media, adherence to diverse substrates, and high electrical conductivity.

**Figure 20:**
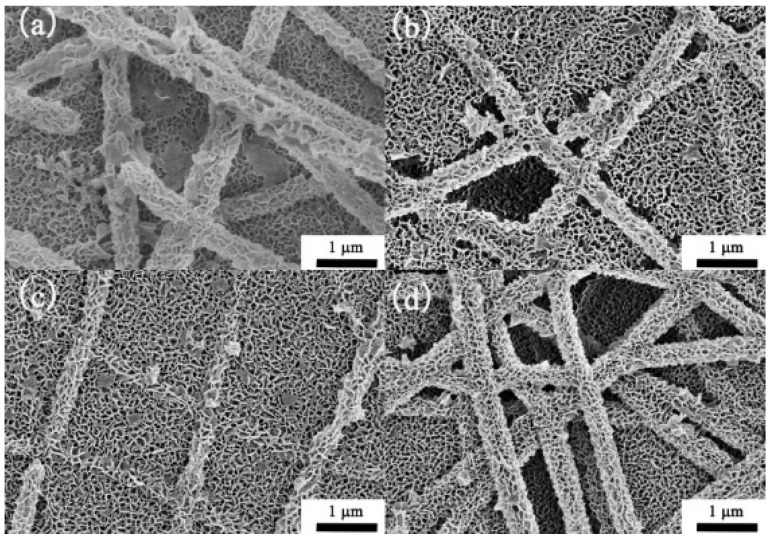
SEM images of SnO_2_/PPy nanocomposite prepared with different doping acids: (**a**) PSSA; (**b**) HCl; (**c**) TSA; and (**d**) CSA (polymerization time: 1 h). (Reprinted/adapted from [258]).

The composite PPy-PVA film showed better sensing performance and rapid response time towards ethanol among other VOC vapors including ethanol, ammonia, chloroform, toluene, and acetone [257]. Among several conducting polymers, PPy has been exploited widely to detect VOC gases at RT [256]. PPy contains several unique and desirable characteristics such as its stability in different environments, high electrical conductivity, ease of deposition from aqueous and non-aqueous media, and its adhesion to various substrates makes it an ideal material for sensing applications that are required for the detection of various kinds of VOCs. With the unique properties of PPy, Li et al. developed a composite of SnO_2_ nanosheet coated with PPy to detect ammonia at RT, as shown in Figure 20 [258]. SnO_2_ is an inorganic semiconductor gas-sensing material that exhibits poor conductivity at RT with no significant response to ammonia gas [259,260]. However, there was a report showing a profound effect on the electrical properties of PPy and its sensing performance towards ammonia. Therefore, in the reported work authors utilized SnO_2_ with PPy to combine the features of both materials to improve the performance of sensors. The nanocomposite sensors exhibited a higher sensitivity of ∼6.2%/ppm for ammonia concentration varying from 1 ppm to 10.7 ppm, with a low LOD ∼257 ppb. The sensor coated with SnO_2_/PPy exhibited a higher response for ammonia compared to the sensor coated with PPy alone.

**Figure 21:**
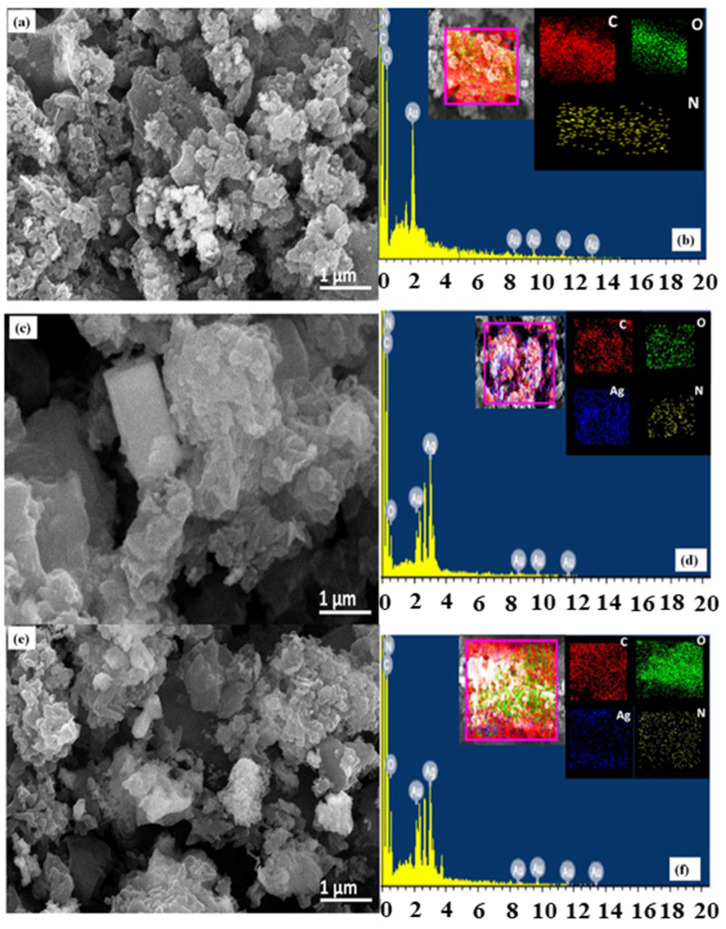
SEM images of (**a**) PPy, (**c**) Ag–Ag_2_O/PPy, and (**e**) Gr/Ag–Ag_2_O/PPy on the scale of 1 μm, respectively; (**b**,**d**–**f**) EDAX spectrum proves the elemental composition of as-synthesized, respectively (inset: presence of C, Ag, N, and O). (Corresponding inset) EDAX map indicates the presence C, Ag, N, and O). (Reprinted/adapted from [263]).

In 2019, Wu et al. reported a PPy-coated polarization-maintaining fiber sensor to detect isopropanol [261]. The sensing configuration was based on the combination of the Vernier effect of a single-fiber Sagnac loop and the isopropanol-sensitive material of PPy. The sensing mechanism is based on the monitoring of the strain effect due to the swelling of PPy in presence of different concentrations of isopropanol. The sensor exhibited a high sensitivity of 239 pm/ppm compared to the conventional Sagnac interferometer in the range of 0 ppm to 42 ppm. Later, in 2020, Setka et al. synthesized Cadmium telluride (CdTe)/PPy nanocomposites and integrated them into love wave (L-SAW) sensors to detect acetone at RT [262]. The sensing response was obtained in terms of frequency shift and calculated by monitoring the change in the resonant frequency in presence of air and the target VOC. The fabricated device exhibited a high sensitivity to acetone among ethanol and toluene along with a lower LOD of 5 ppb. In 2021, Shoeb et al. synthesized Gr/Ag–Ag_2_O/PPy nanocomposites for fast responsive and selective detection of ammonia at RT, as shown in Figure 21 [263]. The sensing response of the device was monitored by measuring the electrical conductivity responses of the device at RT. The composition of Gr/Ag–Ag_2_O/PPy exhibited a 40 times higher amplitude of conductivity variation compared to pristine PPy with the presence of 1000 ppm of ammonia vapor. The sensor also showed excellent selectivity towards ammonia compared to ethanol, acetaldehyde, formaldehyde, benzene toluene, and m-xylene.

**Figure 22:**
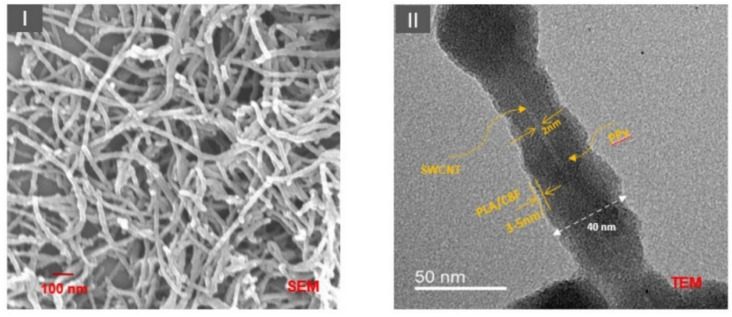
(**a**) The SEM and (**b**) transmission core–shell-shaped nanorods (sample). (Reprinted/adapted from [264]).

Most recently, Byeon et al. synthesized SWCNT/PPy/phenyllactic acid nanocomposite as shown in Figure 22, to detect acetone vapor at RT [264]. The obtained sensing material had structural features similar to heptadecafluorooctanesulfonic acid (C8F)-doped-PPy layer surrounding a single-stranded SWCNT, and the phenyllactic acid layer surrounding the PPy work as a selective sensing layer for acetone. Here, the PLA bonded chemically with the PPy backbone, exhibited a sensing synergistic effect, and greatly improved the sensitivity to acetone. Finally, C8F-doped-PPy/PLA@SWCNT as an acetone sensor also provided reliable detection at a low concentration of 50 ppb at 25 **°C**. Additionally, the fabricated sensor was able to detect a clear signal with the humidity varying in the range of 0% to 80% at a low temperature, which is highly required for medical diagnosis during the breath analysis.

Nowadays, there is a sudden growth in PPy-based VOC sensors due their high sensitivity, selectivity, stability, and rapid response at RT. High sensitivity and significant selectivity sensors can be obtained by integrating or doping the PPy with other materials, e.g., SWCNT, CdTe, SnO_2,_ etc. The only challenging task of the PPy-based VOC sensor is the mixing of PPy with other materials which requires good knowledge of chemistry and expertise in the synthesis.

#### 7.2.2. Polythiophene (PTh)

Among the various conducting polymers, polythiophene (PTh), which is a p-type semiconductor, has been exploited very less for vapor detection compared to the other conducting polymers. However, the trend of using PTh nanocomposites in VOC-sensing technology has been rapidly increasing in the last few decades. In 2015, Tripathi et al. developed PTh/aluminium oxide (PTh/Al_2_O_3_) nanocomposite to study its potential for ammonia gas sensing [265]. The composite was synthesized in pellet form by chemical oxidation technique. The variations in the resistance of the pellets were studied with various concentrations of ammonia within the range of 25 ppm to 650 ppm in the closed chamber. The report showed that the response can be improved by increasing the concentration of Al_2_O_3_ doping as well as increasing the concentration of ammonia. Later in 2016, Bai et al. synthesized hybrid material to detect nitrogen dioxide (NO_2_) utilizing reduced graphene oxide (rGO) and PTh [266]. Graphene has shown great potential in various applications owing to its large surface area, excellent electron transport capacity, and outstanding mechanical strength which was further enhanced by the hybridization with conducting polymers. The results showed that the hybrid material sensor with 5 wt% rGO showed a four times higher sensitivity to 10 ppm of NO_2_ gas and excellent selectivity compared to a pure PTh sensor. Husain et al. reported a series of experiments on PTh and its potential in organic vapor sensing [267,268,269,270]. In 2019, they developed PTh/ZrO_2_ nanocomposite for ethane detection [267]. The synthesized materials were studied for comparative DC electrical conductivity retention under cyclic ageing and isothermal conditions. In this work, the high surface area and efficient conducting network allow the quantitative adsorption and desorption of ethane. Under the ethane gas environment, PTh/ZrO_2_ nanocomposite sensor exhibited a 19 times higher electrical conductivity, representing faster adsorption and desorption towards ethane compared to a pure PTh sensor due to its high surface area. In 2020, they proposed the utilization of PTh/SnO_2_ nanocomposite in a pellet-shaped for alcohol sensing including butan-1-ol (1° alcohol), butan-2-ol (2° alcohol), and 2-methyl propanol (3° alcohol) at RT [269]. The PTh/SnO_2_ pellet exhibited the highest sensing response in terms of the variation in DC electrical conductivity and maximal reproducibility for butan-1-ol. In the same year, Husain et al. developed PTh/GO nanocomposite to detect ethanol vapor at RT [270]. The PTh/GO nanocomposite significantly showed an improvement in DC electrical conductivity for ethanol vapor compared to pure PTh with a low LOD of 400 ppm. From all the research mentioned above, it is clearly observed that an appropriate amount of composite material in PTh may lead to a high sensitivity and selectivity towards the VOC vapors. Although this field is yet to be exploited, it can be widely applied to monitor VOCs at the sub-ppm range.

### 7.3. Molecularly Imprinted Polymer (MIP)

MIPs are polymeric materials synthesized with nanocavities of similar shape and size to the target molecules (also known as templates). The specificity is a distinctive feature of MIPs that makes them an ideal element in the sensing system. In 1894, an idea of molecular interactions was reported by Emil Fischer using the term “lock-and-key” [271]. The first article on the successful imprinting of amino acids in the polymer was reported by Andersson et al. in 1984 [272]. In 1985, the term ‘imprinted polymer’ was first used in history by G. Wulff [273,274]. He proposed the covalent-binding technique to create the imprinted polymer. The MIPs can be synthesized for almost every kind of target molecule despite its shape, size, and chemical structures. Generally, the synthesis of MIPs follows a similar outline as follows [275] (i) combination of monomers with template (ii) polymerization under UV or hot air (iii) extraction of template from the polymer to create a specific cavity for binding with target molecules. With the ability to do so with every kind of target molecule, MIP plays an outstanding role as an analytical tool in the development of tailor-made sensors and gains huge interest in VOC detection [276,277]. The MIP-based sensors exhibit a full range of properties including high selectivity, sensitivity, great reliability, low cost, and mechanical, thermal, and chemical stability [278].

**Figure 23:**
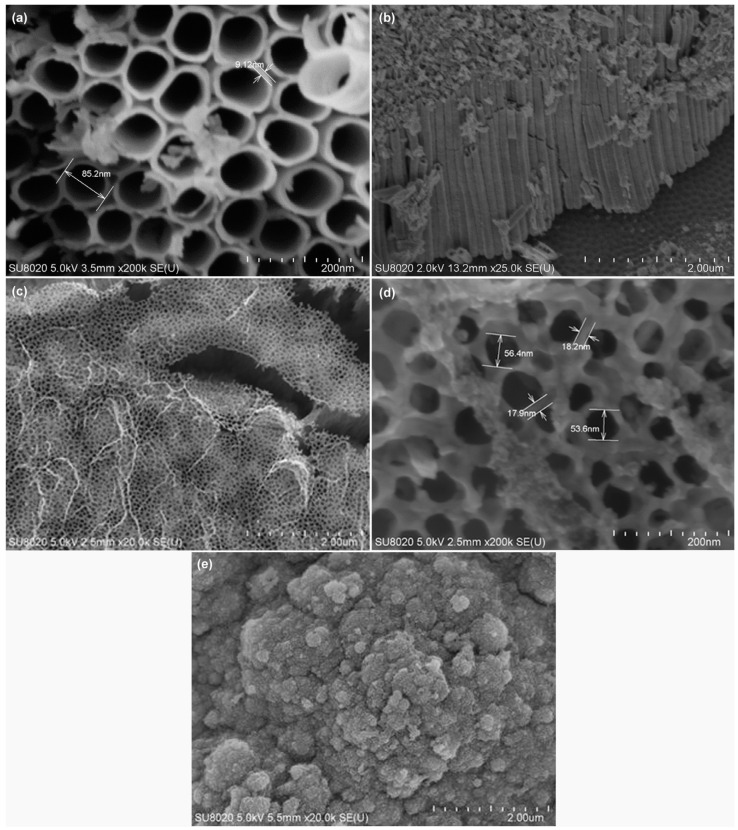
SEM images of (**a**) top view of TiO_2_ nanotube array, (**b**) cross-section view of TiO_2_ nanotube array, (**c**) thin layer of molecularly imprinted polypyrrole synthesized on a TiO_2_ nanotube array, (**d**) zoom of (**c**); and (**e**) thick polypyrrole film on the flat substrate (Reprinted/adapted from [281]).

In 2014, Mustafaa and Lieberzeit reported a MIP/Ag_2_S nanoparticle composite coated on quartz crystal microbalance (QCM) for aliphatic alcohol detection [279]. Mixing MIP with Ag_2_S nanoparticles results in a nanocomposite material with the collective properties of both materials. The MIP/Ag_2_S nanocomposite gave a normalized response of −70 Hz for 1-butanol vapor at 400 ppm, which was two times higher response compared to the pure Ag_2_S layer of similar thickness. The synergy produced by the strong affinity of Ag_2_S nanoparticles and the selective ability of MIPs toward 1-butanol vapor allowed fabricated sensors to have a relatively low detection limit (LOD) of 2 ppm. Later in 2016, Hussain et al. reported the QCM sensor coated with MIP for the detection of formaldehyde vapors in air streams [280]. The sensor achieved a low LOD of 500 ppb with good selectivity for formaldehyde compared to methanol, acetaldehyde, formic acid, and dichloromethane. In 2017, Tang et al. proposed another sensor for formaldehyde detection utilizing MIP/TiO_2_ nanotube array composite, as shown in Figure 23 [281]. In this work, the MIP/TiO_2_ nanotube array had a high surface-to-volume ratio, resulting in the improvement of sensing performance. As the humidity is neglected, the sensor can selectively detect formaldehyde in the ppm range at RT along in the long term.

**Figure 24:**
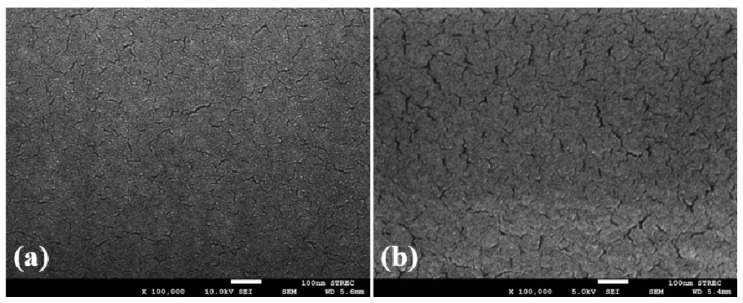
(**a**). Non-imprinted polymer (**b**). IPA-MIP surface. (Reprinted/adapted from [283]).

In 2019, Abdelghani et al. developed highly sensitive SnO_2_ nanostructured electrochemical sensors utilizing MIP technique to detect acetone and ammonia, simultaneously [282]. Four SnO_2_ films were prepared using a spin coater under various conditions of introducing acetone and ammonium hydroxide as template molecules during the hydrothermal synthesis. A 500 nm thicker regular micro sheets have been developed from SnO_2_ nanoparticles of various sizes ranging from 20 nm to 50 nm (based on the synthesis condition). The sensing response of the device was tested against liquid petroleum gas (LPG), ammonium hydroxide, oxygen, acetone, and benzene. The result showed an increase in response for ammonia and acetone with the increment of the participation of the solvent during the synthesis. The results exhibited a promising response for ammonia gas with a highest sensitivity of 89% at 260 °C while for acetone the sensor exhibited a sensitivity of 77% at 230 °C. In a recent study published in 2022, Pathak et al. reported an optical sensor utilizing a MIP layer on a glass slide to detect isopropanol (IPA) vapor [283]. The work illustrated the surface morphology difference between non-imprinted and imprinted polymers, as shown in Figure 24. The regular texture for non-imprinted polymer appeared because of the absence of the IPA template during the synthesis leading to no specific binding sites for IPA molecules. However, the crack and/or porosity appeared on the imprinted polymer surface due to the successful removal of the IPA templates. The increase in the wrinkled structure and surface roughness in the imprinted polymer can be attributed to the increase in the surface area due to the accommodation of the IPA template. The fabricated device was investigated over IPA solutions with various concentrations varying from 50% to 100%. The sensor exhibited a maximum sensitivity of 0.63 nm/%IPA vapor at 120 min exposure, along with good selectivity among a similar class of VOCs.

In the same year, Mousazadeh et al. reported a chemorestive sensor with the use of Au/MIP nanocomposite to detect hexanol, which is a cancer biomarker [284]. The composite-sensing material was coated on an interdigitated electrode. The variation in electrical response and resistance of the device was analyzed by Fourier transform cyclic voltammetry with the presence of various concentrations of hexanal gas. The sensor exhibited a low LOD of 1.1 ppm for a linear range of hexanal concentration varying from 2.5 ppm to 300 ppm. Additionally, the device showed three times higher sensitivity to hexanal compared to the other VOCs with similar carbon atom numbers, exhibiting a superior selectivity sensor for hexanol.

### 7.4. Advantages and Limitations of Polymer-Based VOC Sensors

CP-based VOC sensors are nanomaterial-based sensors. This technology is commercialized under the name Cyranose 320, which comprises an array of 32 polymer composite sensors [285]. Although the CPs are highly sensitive to several VOCs along with the rapid response time, it is also sensitive towards humidity and temperature. Therefore, the device suffers from cross-sensitivity leading to an inaccurate readout. Whereas, the imprinted polymer is considered a promising candidate for selective detection applied under a mixed gaseous environment. However, the response time of the MIP sensors is longer due to the difficulty associated with diffusion [286]. The sensitivity and efficiency of MIP-based sensors are directly related to the number of imprinted cavities available on the sensor surface.

In Table 5 we have summarized the various kinds of metal oxide sensors and their sensing performances.

## 8. Other Sensing Materials

Aside from these renowned sensing materials, some other materials have also been exploited by researchers due to their superior adsorption capability towards the VOC gasses. Some of them are selected to be discussed in the following section due to their excellent performance which can be promising in the VOC-sensing area.

### 8.1. Metal Organic Frameworks (MOFs)

MOFs have emerged as an excellent sensing material for gas sensing due to its crystalline nature, extreme porosity and high surface areas [305]. These properties allow the MOFs to have the ability to sense both the inorganic and organic compounds. The MOFs used for the adsorption of VOCs are mainly Material of Institute Lavoisier (MILs) series and isoreticular metal-organic frameworks (IRMOFs) series which are discussed in detail in the following subsections. Over the past two decades, the MOFs have been exploited well and currently, there are more than twenty thousand MOFs materials available [306,307,308].

#### 8.1.1. Material of Institute Lavoisier (MIL) Series

MIL-n is a series belonging to the class of materials in MOFs which was first synthesized by Ferey’s group [309]. The synthesized MIL-101 comprised a mesoporous structure with a pore diameter of 2.9 nm to 3.4 nm and an internal surface area of 6000 m^2^/g [309]. Due to the porous structure of MIL-101, it gains a huge interest in gas-sensing applications [310]. In 2011, Huang et al., firstly, developed a QCM-based VOCs sensor utilizing MIL-101 [311]. They reported that MIL-101 exhibits various affinities for the various VOCs molecules due to the energy heterogeneity of MIL-101. The adsorption isotherms of the VOCs on MIL-101 followed the Dubinin–Astakhov equation with the characteristic energy from 5.70 (methanol) to 9.13 kJ.mol^−1^ (n-butylamine), Astakhov exponent from 0.50 (n-butylamine) to 3.03 (n-hexane), and the limiting adsorption capacity from 0.08 (n-hexane) to 12.8 (n-butylamine) mmol.g^−1^. MIL-101 showed the weakest affinity for n-hexane and the strongest affinity to n-butylamine. The dynamic adsorption mechanism mainly relies on the metal sites of MIL-101 which leads to greater VOC adsorption capacity and higher affinity compared to the AC. Most recently in 2022, Mousavi and Zeinali reported VOC detection based on resistive gas nanosensor using MIL-101(Cr)/CNT nanocomposite [312]. The sensor was investigated over ethanol, methanol, isopropanol, formaldehyde, acetonitrile, acetone, tetrahydrofuran, dichloromethane, and n-hexane at RT. The sensor exhibited an excellent selectivity towards formaldehyde along with a low LOD of 0.07 ppm, and high sensitivity of 325.21%. The response time of the sensor was 6 min over a wide VOC concentration range varying from 10 ppm to 1000 ppm. The molecular size, chemical structures, and polarity of the material were the key factors which affect the sensing performance of the device.

Although the MIL-n series shows an excellent performance in gas separation and adsorption, there are still several limitations including poor stability, insufficient storage capacity, and high cost leading to limited research exploitation possibilities. However, the specific surface area, metal sites, pore structure, π-π binding, and modifiable groups are some of the key parameters which can affect the separation and adsorption processes of different VOCs. Currently, researchers are interested in designing and improving the structure of the MOFs materials, in order to develop a new MOFs material to attain specific functions and overcome their limitations.

#### 8.1.2. Isoreticular Metal-Organic Frameworks (IRMOFs) Series

IRMOFs are a series of MOFs that own similar network topologies. Introducing a simple substitution of organic linkers of IRMOF-1 (i.e., MOF-5), other IRMOFs can be produced which shows unique property such as high chemical stability and a large Brunauer, Emmett and Teller (BET) surface areas [313]. In 1995, Yaghi et al. synthesized micropores MOF to selectively bind the organic guest molecules [314]. The building block was a symmetric organic molecule, which selectively binds metal ions in order to form a layer of the metal organic compound. This layer was alternatively formed with the layers whose composition was determined by the functionalization of the starting molecules creating channels which allow the selective binding of guest molecules. The crystal lattice of IRMOFs layers was stable at 350 °C even after the removal of the guest aromatic molecules and it selectively adsorbs gaseous molecules. The stability and rigidity of IRMOFs materials allow the size and shape-selective absorption of organic molecules and ions.

In 2015, Yaghi et al. proposed a high-absorptive material named MOF-5 (IRMOF-1) from benzene and octahedral Zn-O-C clusters links [306]. In this work, the author also showed that the synthesized porous material can be functionalized with organic groups such as NH_2_, Br, OC_3_H_7_, C_2_H_4_, OC_5_H_11_, and C_4_H_4_. In addition, its pore size can be expanded with a long molecular structure such as tetrahydropyrene, terphenyl, pyrene, and biphenyl. In order to improve the adsorption performance, pore texture and crystal structure of MOF-5, Saha et al. utilized diethylformamide as a solvent in the synthesis instead of the conventional solvent [315]. This synthesized MOF-5 comprised a large specific surface area of 3917 m^2^/g, with a pore size of 8.6 Å and pore volume of 1.39 cm^3^/g, respectively. Yang et al. developed MOF-177 at 85 °C by using solvent thermal technique and exhibited its superior adsorption capacity for VOC molecules [316]. In the reported work, the specific surface area of MOF-177 was 4170 m^2^/g, with an average pore size of 0.94 nm, and pore volume of 1.11 cm^3^/g, respectively. The material exhibited great adsorption towards benzene, toluene, acetone, ethylbenzene, o-xylene, m-xylene, and p-xylene in the air. From the above investigation, it is quite clear that the MOFs show an excellent adsorption capacity due to their large specific surface areas and may become good candidate materials in the VOC sensing.

### 8.2. Zeolites

Zeolites are micropores crystalline aluminosilicates with a highly ordered porous structure. Zeolites are widely utilized in various sensing applications including heavy metal detection [317], biosensing [318], VOC gas sensing [310] due to their tunable pore size, acceptable thermal stability, high hydrophobicity, and ease of modification of their surface. According to the previous research work, the average specific surface area of zeolites has been determined to be 800 m^2^/g, which can be maximized up to 1769 m^2^/g [215].

**Figure 25:**
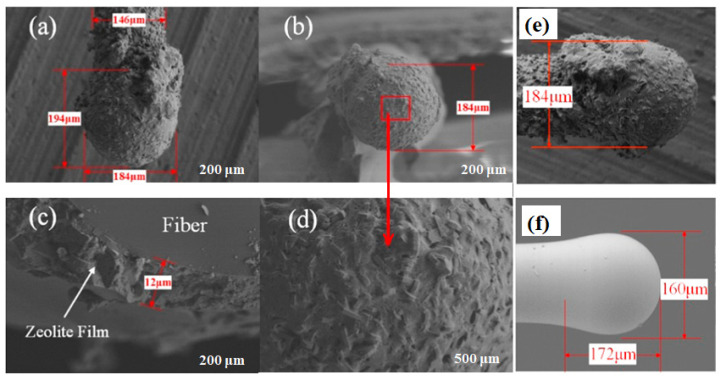
Zeolite film-coated fiber. (**a**). side view, (**b**). front view the, (**c**). cross-section, (**d**): enlarged view, (**e**). zeolite layer on fiber, and (**f**). spherical-shaped fiber end. (Reprinted/adapted from [320]).

In 2009, Bhatia et al. reported the adsorption of butyl acetate using silver-exchanged yttrium (AgY (IE)) and silver-loaded zeolites (AgZSM-5 (IE)) at 28 °C [319]. The Ag was loaded in the zeolites via ion exchange and impregnation techniques. The adsorption capacity of AgY (IE) against the butyl acetate was reduced by 42% due to the increase in the RHof the VOCs. However, the adsorption capacity of AgZSM-5 (IE) for butyl acetate was negligibly affected by the humidity, and the adsorption capacity was only reduced by 7%. In 2016, Xiangping et al. utilized the optical fiber as the sensing platform for the detection of VOC traces [320]. In the reported work, the author utilized the feature of nanoporous zeolites for the selective adsorption of isopropanol. The device comprised of the spherical-shaped fiber coated with thin zeolite film at the edge, as shown in Figure 25a–d. The zeolite layer and spherical shaped fiber formed an arc-shaped inline FP cavity, which improved the sensing performance, shown in Figure 25e,f. The proposed sensor exhibited enhanced sensitivity of 0.91 nm/ppm for IPA concentrations ranging from 0 ppm to 70 ppm.

An electrochemical sensor developed from SnO_2_/ZSM-5 zeolite for the detection of formaldehyde was reported in 2021 by Sun et al. [321]. The composite material was highly selective and sensitive towards the formaldehyde due to the large number of oxygen vacancies existing on the surface of the composite allowing the rapid adsorption of formaldehyde. The result obtained from SnO_2_/ZSM-5 composite had a better sensing performance compared to the pure SnO_2_. This improvement was attributed to the composite structure by integrating ZSM-5 zeolite.

Zeolites are generally used in commercial separation processes and VOCs recovery due to their molecular sieving ability to preferentially adsorb certain molecules and to exclude others [322]. The size and the structure of both the micropores and the molecular adsorbate determine the selectivity and adsorption capacity. The sorbed VOC molecules may interact with (a). the zeolite lattice, (b). extra-framework cations, and (c). other sorbed molecules. The polarity of the probe molecule, its dimensions, and its partial pressure define the kind and the magnitude of interaction with a given adsorbent. On the other hand, for a given adsorbate, the number and the place of the free cations, the topology, the size of the channels, and the possible pre-adsorption of other molecules (e.g., humidity) also affect this adsorbate/adsorbent interaction. MCM-41 and SBA-15 are the types of zeolites which are widely used in VOC detection [323,324].

## 9. Future Prospects

This review highlights the potential use of active materials including metal oxides, polymers, conductive polymers, carbon-based materials, composites, and other adsorption materials as a VOC biomarker sensor due to their excellent sensing property and wide applicability. These materials are representatives for sensing clinical VOC biomarkers. The enhanced properties of these materials such as sensitivity, thermal stability, conductivity, selectivity, and fast responses generally include the control of their composition, size, and morphology. Some of the modified structures of these materials such as hierarchical structures, bilayer structure formation by the introduction of tunable attractive elements, such as by doping, have displayed an efficient improvement in VOC-sensing performances. VOC has increasingly affected the ecological environment and human health due to its high toxicity, carcinogenic, and photocatalytic in nature. Therefore, there is a need for their precise detection, monitoring, and controlling adverse effects of these VOCs effectively by combing the aforementioned materials in real time. Metal oxides such as ZnO, NiO, TiO_2_, and MoO_3_ offer admissible responses to a different variety of VOCs due to their nature of absorbing gases/vapors and converting them into conductivity variations. Moreover, the usage of carbon-based materials, polymers, and other adsorption materials also have similar properties to metal oxides such as charge transfer between the gases/vapor species, oxidation, and the reduction of the dopants (n- or p-type) which are widely accepted as a promising candidate in the VOC-sensing applications. Their different modified morphologies at the nanometric scale with the introduction of different tunable elements have attracted the attention of many researchers because of their increase in a surface-to-volume ratio that gives rise to high sensitivity, fast response speed, lower operating temperatures, and good stabilities. However, the advances in terms of selectivity in a mixed volatile environment still remain to be a challenging task.

Although outstanding advantages were achieved by implementing those materials into the VOC sensors, there are still major challenges involved in the clinical diagnosis of the VOC biomarkers. The thorough investigation in the functionality of materials to a larger group of VOCs as well as the implementation of other sensing mechanisms such as optical, electrical, and mechanical-based gas/vapor sensors are in high demand. In terms of materials, the accurate detection, and the sensitivity for the low concentrations (ppb-ppt levels) of the VOC biomarkers is a prime concern. Therefore, there is a strong need in exploring and utilizing new hybrid materials, i.e., (i) by the combination of metal oxide and carbon-based materials, (ii) metal oxide and polymers, and (iii) carbon-based materials and polymers towards VOC detection to overcome the issue of poor reproducibility in routine use. It is also worth mentioning that by combining varieties of these materials, it is expected to provide promising results when operating them together, thus offering strong stability in abrasive variations of humidity and/or temperature.

Further, numerous reports can be found on the use of doping and composite materials for tailoring the sensor responses towards specific VOCs. However, valid information on efficacy and the role of such materials of VOC detection are limited. Moreover, a large number of reports are available for chemoresistive sensors that work under the resistive principle but not on electrical, optical, and mechanical detection of VOCs. Thus, in the future, the properties of materials for VOC detection should be explored more by utilizing the various physical changes. As these physical changes in the properties of materials may have the possibility to offer better selectivity for VOC-sensing devices.

In summary, it can be seen that various challenges and concerns associated with VOC sensors are mostly based on the type of materials synthesis method discussed in this review. From the materials discussed earlier, incorporating them into developing gas sensors for the detection of associated clinical VOC biomarkers is known to influence its sensing performances. These materials are therefore gaining a huge interest in designing clinical and diagnostic tools.

Thus, the clinical use of the VOCs from the human exhaled breath can be implemented by developing a versatile breath gas sensor with specific requirements depending on the applications to achieve a high response value, exceptional selectivity, lower LOD, great stability/reproducibility, stable calibration of the sensor system upon malfunction and high response/recovery speeds with a lower detection limit in real time. Moreover, from the economic point of view, these gas sensors based on the materials discussed should be universally available and should provide ease of fabrication, wider compatibility, and simple and low cost for easy implementation in different transducing platforms.

## 10. Clinical Challenges

Human breath analysis is an interdisciplinary field of research work which includes analytical techniques, data processing, medical science, materials chemistry, and engineering. Health monitoring and disease detection via blood analysis are invasive techniques which are painful to the patient. In the past decades, exhaled breath analysis has emerged as a better alternative to diagnose the disease at an early stage because of its non-invasive nature. However, the method is fraught with several challenges. Direct exhaled breath cannot be used in all breath analysis methods. Breath collection and its storage became a major issue. The collected breath which is stored for a long time is often degraded and hence, their original compositions are changed. Researchers are still not sure whether mouth-exhaled or nose-exhaled breath should be utilized for the VOC analysis. Currently, it is advised that dead space air should not be considered for breath analysis; only end-tidal breath should be used. However, for highly water-soluble breath biomarkers, such as isoprene and acetone, there is no anatomic dead space to be defined. Most of the sensor responses are dependent on the exhaled breath flowrate onto the sensor head. In the direct breath analysis, patients are allowed to directly exhale onto the sensing head. However, different patients would exhale at different flow-speeds, leading to inconsistent measurements. The exhaled breath concentration of various VOC gases varies significantly according to the patient’s gender, age, weight, race, food habits, pregnancy, lifestyle, etc. In addition, not all exhaled breath components are endogenous; rather, most of them are exogenous in origin. Many of the endogenous gases are not even systemic in origin. Therefore, locality becomes another confounding factor. In this section, a comparison of the performance is drawn between the gold-standard devices and other devices based on non-biological sensing materials. The selective detection of diseases from exhaled breath is limited to a small number of diseases, e.g., chronic obstructive pulmonary disease, diabetes, etc., which can be monitored using a single biomarker. In most diseases, especially various cancers, the concentrations of breath VOCs varied simultaneously. Therefore, the use of target-specific detection demands the development of highly selective sensing materials which is a paramount challenge in itself. Other sensors with poor selectivity can suffer problems from low to medium sensitivity. Therefore, obtaining sufficient discrimination between diseased and healthy groups might become difficult.

Gold standards: GC–MS, SIFT-MS, and PTR-MS are the most accurate detection techniques for exhaled breath analysis with an analysis time of millisecond. These instruments require a fraction of ml of liquid or gas samples for monitoring. These classical instruments are expensive and require a trained person for handling, data analysis, and data interpretation. These instruments are not like any household devices and can only be available in hospitals and diagnostic clinics. This impedes the use of these instruments as breath analyzers on a day-to-day basis at our homes. One of the major goals of breath analysis is early detection of disease by regular health monitoring and for that to be possible the device used must be financially affordable for the common man, easy to use even for a layman and handheld.

MO sensing materials: MO-based VOC sensors are cost-effective, easy to use, compact and capable of detecting several VOC biomarkers at ppm, ppb, and even, at ppt levels. Although most MO sensors exhibit excellent performance, a lack of specificity limits them to be used as a medical diagnostic tool. MO-based VOC sensors are also sensitive to humidity. Human breath contains almost saturated moisture which acts as a major cross-sensitive agent impeding the response of the sensor to target VOC molecules. Currently, thick film MO sensors are available in the market. The thick MO film-based sensors suffer high power consumption and poor sensitivity due to a low diffusion rate. MEMS-based sensor arrays can resolve the issue of power and size to some extent; however, they comprise other problems. The microsystems generally utilize moisture traps, which adsorb moisture along with the target gases. Humidity dependence of gas adsorption can significantly reduce the reliability and reproducibility of the pre-concentrator. Pre-concentration requires longer time; thereby, making real-time analysis difficult. Further, the small volume of pre-concentrator materials present in such microsystems might not be sufficient for the detection of trace gases. Selective and reversible pre-concentration remain challenges.

Carbon-based VOC sensors: By coupling MO, e.g., ZnO, NiO, MoO_3_, and TiO_2_ with innovative carbon-based sensing materials such as GO, rGO, MWCNTs, AC, etc., the performance of the device can be improved in terms of sensitivity, LOD, response and recovery times towards the specific gaseous targets. Although most of the sensors show excellent performance, many of them fail to fulfill the requirement of medical diagnosis due to poor selective performance in a mixed gaseous environment. Even though GO, CNT, AC, etc., are considered novel VOC-sensing materials, they still require further study and improvement for commercialization.

Polymer-based VOC sensors: Polymer-based VOC sensors are considered selective tools for breath biomarkers detection. Among various polymers, the MIP can be considered an excellent material to selectively detect VOC in very low-level concentrations of ppm/ppb. Although the MIP-based sensor provides a good selective response towards the target gas it suffers from the issue of rapid response time. The field is still growing; therefore, the commercialization of polymer-coated VOC sensors has not been explored yet.

## 11. Conclusions

It has been reported previously that each human generates unique VOC profiles in their exhaled breath which can be utilized as biomarkers in order to monitor and diagnose various disease conditions. In the past decades, several sensing materials have been reported to achieve high selectivity and sensitivity in the sub-ppm range. In this review, a detailed literature survey of the recent progress of VOCs sensing materials and the key factors to improve the sensing response and to make it reliable for real-time utilization are provided. Each sensing material comprises some advantages and limitations which are briefly outlined at the end of each subheading. The new idea and approaches to improve the sensing performance of materials concerning adhesion, stability, and selectivity are also suggested.

## Figures and Tables

**Table 1:** Summary of most important VOCs and associated disorder along with their concentrations.

Exhaled VOC	Molecular Formula	Associated Disorder	Concentration (Healthy Person)	Concentration (Diseased)	Refs.
Isoprene	C_5_H_8_	Diabetes, heart failure, lung cancer	5.8 ppb to 274.9 ppb		[19,20,21,38]
Acetone	C_3_H_6_O	Diabetes,	0.39 ppm to 0.85 ppm	T1D: >2.2 ppm, typically >10 ppm	[22,39]
Ammonia	NH_3_	Liver dysfunction, kidney failure, hepatic encephalopathy, type-II Alzheimer, swelling of the brain	0.25 ppm to 2.9 ppm	NA	[25,26,40]
Methane	CH_4_	Oxidative stress, heart disease, breast cancer	4 ppm to 10 ppm	8 ppm to 50 ppm	[41,42]
Aldehyde	R−CH=O	Liver cancer, Alzheimer, Parkinson’s Disease	13 ± 5 ppb	NA	[41,43]
Hydrogen sulfide	H_2_S	Asthma, oral, and dental health	8 ppb to 16 ppb	NA	[41,44]
Nitric Oxide	NO	Asthma, acute lung, injury, lung infection, lung cancer	<35 ppb	NA	[45,46]
Ethane	C_2_H_6_	Scleroderma, cystic fibrosis	0 ppb–12 ppb	NA	[47,48]
Pentane	C_5_H_12_	Myocardial infarction	0.3 nmol/L to 0.8 nmol/L	NA	[49]
Carbon monoxide	CO	Smoking	0.4 ppm to 0.8 ppm (non-smokers)	2 ppm to 20 ppm	[50,51]
8-isoprostane	-	Obstructive sleep apnea	4 pg/mL to 5 pg/mL	6.7 to 7.1 pg/mL	[52]
Interleukin-6	IL-6	Non-small cell lung cancer	3.3 pg/mL to 3.7 pg/mL	9.3 pg/mL to 9.9 pg/mL	[53]

**Table 2:** Advantage and limitations of classical detection techniques.

Technique	Advantage	Limitations
GC–MS	Identification of organic components through separating complex mixtures Quantitative analysis Trace detection of organic contamination as low as ppb level for liquid matrices and nanogram level for solid matrices	Non-volatile matrices require additional preparation such as extraction, outgassing, etc. Target compounds must either be volatile Atmospheric gases such as N_2_, CO_2_, O_2_, CO, Ar, H_2_O are challenging
PTR-MS	Quantitative monitoring Absolute concentrations can be determined without previous calibration Fast analysis, i.e., within milliseconds and online, which allow the real-time monitoring of exhaled breath	Only molecules with a proton affinity higher than water can be detected Cannot provide as much information as GC–MS
SIFT-MS	Quantitative monitoring Absolute concentrations can be determined without previous calibration Fast analysis, i.e., within milliseconds and online, which allow the real-time monitoring of exhaled breath	This technique uses three reagent ions, i.e., NO^+^, H_3_O^+^, and O^2+^, which makes it suitable for detection of volatiles with lower proton affinities than water. Cannot provide as much information as GC–MS

**Table 3:** Summary of all MO based VOC sensors.

Material	Morphology	VOC	Sensing Range	Response Time	Operating Temperature	Refs.
**ZnO based VOC sensors**						
ZnO	Flower-like microstructure	Ethanol	50 ppm	12 s	RT	[161]
ZnFe_2_O_4_/ZnO	Flower-like microstructures	Acetone	50 ppm	2 s	250 °C	[162]
ZnO/CuO on graphene substrate	Nanoflower	Ammonia	5 ppm	4.1 s	RT	[163]
ZnO QDs	Nanoparticles	Isoprene	1 ppm	8 s	150 °C	[164]
ZnO@CuO	Porous hollow sphere nanoparticle	H_2_S	10 ppm	33 s	RT	[165]
ZnO/Zn_2_SnO_4_	Microflowers	CH_4_	400 ppm	10 s	250 °C	[166]
SnO_2_/ZnO	Nanosphere	Formaldehyde	20 ppm	27 s	200 °C	[167]
ZnO/Au	Porous nanosheets	NO_2_	1 ppm	1 s	RT	[168]
ZnO	Thin film	NO_2_	100 ppm	3 s	200 °C	[169]
**NiO based VOC sensors**						
NiO:Al	Thin films	Hydrogen	100 ppm	388 s	RT	[170]
		Methane	100 ppm	1737 s		
NiO/ZnO	hollow microspheres	Toluene	100 ppm	2 s	300 °C	[92]
NiO-In_2_O_3_	nanosphere	N-propanol	100 ppm	541 s	210 °C	[171]
		Formic acid	100 ppm	215 s	200 °C	
		Ethanol	100 ppm	263 s	240 °C	
		Methanol	100 ppm	38 s	300 °C	
		Isopropanol	100 ppm	139 s	250 °C	
		Acetone	100 ppm	141 s	250 °C	
NiO	Mesoporous	Ammonia	0.4 ppm	54 s	RT	[91]
**MoO_3_ based VOC sensor**						
Pt-MoO_3_	Nanobelts	Formaldehyde	200 ppm	8.8 s	27 °C	[172]
α-MoO_3_	Nanorod	Methanol	500 pm	32 s	300 °C	[173]
α-MoO_3_	Nanobelts	Ethanol	500 ppm	14 s	300 °C	[174]
Zn-MoO_3_	Nanobelts	Ethanol	250 ppm	121 s	240 °C	[175]
Fe_2_O_3_/MoO_3_	Nanobelts	Xylene	100 ppm	4 s	233.5 °C	[176]
Ni-MoO_3_	Nano-pompon	Xylene	100 ppm	1 s	250 °C	[177]
**TiO_2_ based VOC sensors**						
TiO_2_ (rutile)	Thin film	NH_3_	12.5–100 ppm	2 min	210 °C	[178]
α-Fe_2_O_3_-TiO_2_	Heterostructure Thin film	Ethanol	100 ppm	46 s	325 °C	[179]
TiO_2_-WO_3_	Thin film (Nanoparticles)	Xylene	10 ppm	410 s	160 °C	[180]
Ag/TiO_2_	Nanoparticles	Acetone	100 ppm	11 s	275 °C	[181]
Fe/TiO_2_	nanotubes	H_2_S	50 ppm	22 s	100 °C	[182]
TiO_2_-ZnO core shell	nanorods	N-butanol	100 ppm	17 s	200 °C	[183]
F/TiO_2_	nanosheets	Acetone	400 ppm	162 s	RT	[184]
TiO_2_–SnO_2_	nanofibers	Formaldehyde	100 ppm	13 s	RT	[185]

**Table 4:** Summary of all carbon-based VOC sensors.

Material	Morphology	VOC	Sensing Range	Response time	Operating Temperature	Refs.
**GO-based VOC sensors**						
ZnO/GO	Nanorods/Sheet	Acetone, Benzene, Ethanol, Methanol	10 ppm to 500 ppm	8.79 s to 12.43 s	200 °C to 500 °C	[225]
rGo/NiO	Sheet/ Hierarchical Flower-like	Methanol	61.51 ppm to 100 ppm	16 s to 30 s (Graph Estimate)	180 °C	[191]
PEDOT: PSS/GO	Nanowire	Acetone, p-Xylene, Ethanol, n-Hexane	5 ppm to 25 ppm	8 s to 12 s	RT	[226]
CuO/rGO	Sheet/ Hierarchical	Ethanol	5 ppm to 200 ppm	23 s	250 °C	[192]
SnS_2_/rGO	3D Porous Structure	Nitrogen Dioxide	0.5 ppm to 8 ppm	153 s	RT	[194]
SnO_2_/GO	Nanocomposite Heterojunction	Ethanol, Acetone, Ethylbenzene	1 ppm	120 s	RT (With UV light) 300 °C	[227]
SnO_2_/rGO	Nanorods Array/3D Hierarchical Structure	Formaldehyde	10 ppm to 50 ppm	52 s	50 °C to 150 °C	[195]
ZnO-SnO_2_/rGO	Ternary Hybrid Nanocomposite with p-n-n heterojunction	Acetone, Ethanol	1–10 ppm	10 s	150 °C	[198]
**MWCNT-based VOC sensor**						
WO_3_/MWCNTs	Nanobrick thin film	Ammonia	60 ppm	512 s	RT	[228]
PDDA/MWCNTs	Thin film	Carbon monoxide	20 ppm	18 s	RT	[229]
ZIF-8/MWCNTs	Sharp facets crystals thin film	Formaldehyde	5 ppm	12 s	RT	[230]
Al_2_O_3_/MWCNTs	Porous film	Carbon dioxide	450 ppm	53.7 s	RT	[231]
In_2_O_3_/MWCNTs	Nanobar SAED pattern	Acetone	250 ppm	9 s	300 °C	[232]
PPy/N-MWCNTs	Loose granular structure	Nitrogen dioxide	5 ppm	65 s	RT	[233]
PANI/MWCNTs/MoS_2_	Porous film	Ammonia	0.25 ppm	32 s	RT	[234]
PVA/MWCNTs	Entangled porous, thin film	Ethanol	100 ppm	24 ± 3 s	RT	[235]
PANI/MWCNTs	Entangled porous, thin film	Ammonia	2 ppm	6 s	RT	[236]
NiO/MWCNTs	Aggregated flake-like structure	Ethanol	100 ppm	27 s	180 °C	[237]
**AC-based VOC sensors**						
AC	Non uniform sheet-like structure	Isopropanol	1 µL to 10 µL	100 s	RT	[238]
Ag-AC-PEDOT: PSS	Nanoparticle composite conductor	Ethanol	(a) 250 mTorr (b) 500 mTorr (c) 1000 mTorr	(a) 470 s (b) 610 s (c) 609 s	RT	[239]
AC	Pistachio shell base	Benzene	700 ppm and 1000 ppm	NA	30 °C to 45 °C	[240]
Granular AC	NA	Methanol, Butanone, Benzene, Ethanol, n-Propanol, o-xylene, Toluene	6000 ppmv	NA	RT	[241]
TiO_2_/AC fiber felt	Porous composite structure	Toluene	<1150 ppm	NA	RT	[242]

**Table 5:** Summary of all various polymers-based VOC sensors.

Material	Morphology	VOC	Sensing Range	Response Time	Operating Temperature	Refs.
**PDMS-based VOC sensors**						
PDMS	Film	Toluene	0.833 g.m^−3^ to 100 g.m^−3^	5 s	RT	[252]
PZT-PDMS	Composite film	Acetic acid Toluene	45 ppm to 100 ppm 0.2 ppm to 0.6 ppm	50 s	RT	[254]
PDMS	Film	Isopropanol	0 ppm to 500 ppm	50 s	RT	[253]
PDMS and PDMS-PDPS	Film	m-xylene and cyclohexane	0 ppm to 1000 ppm 0 ppm to 3500 ppm	150 s	RT	[287]
PDMS and SU-8	Film	Toluene acetone	0 ng to 20 ng 0 ng to 150 ng	0.9 s 0.5 s	RT	[288]
PDMS	Film	Alcohol, ethers, alkanes	0 ppm to 24,000 ppm	240 s	RT	[289]
**Polypyrrole-based VOC sensors**						
PPy loaded Sn_1−x_Sb_x_O_2_	Nanocubes	Ammonia	0 ppm to 20 ppm	~4 s	RT	[290]
PPy	Single nanowire	Heptanal, Acetophenone, Isopropyl Myristate and 2-Propanol	0.3% to 5%	˂200 s	NA	[291]
PPy/MoO_3_	Film	Formaldehyde and acetaldehyde	100 ppm	140 s	RT	[292]
PPy doped dodecylbenzene sulfonic acid	Film	Methane	20 ppm	NA	RT	[293]
Au/PPy	Nanorods	Acetic acid, Benzene, toluene	10 ppm to 100 ppm	20 s	NA	[294]
PPy	Nanoparticles	Methanol, acetic acid, Acetonitrile	NA	˂1 s	RT	[295]
**Polythiophene-based VOC sensor**						
PTh/MoO_3_	Sheet/Rectangular particle	Ammonia, methanol, acetone	1 M concentration	NA	RT	[296]
PTh/Al_2_O_3_	Nanocomposite	Ammonia	25 ppm to 650 ppm	25 s to 72 s	RT	[265]
rGO-PTh	Hybrid Film	Nitrogen oxide	10 ppm	26.36 s	RT	[266]
PTh/SnO_2_	Nanocomposite	Nitrogen oxide	10 ppm to 200 ppm	Few min	RT	[297]
PTh/ZrO_2_	Nanocomposite	Ethene	NA	NA	RT	[267]
PTh/WO_3_	Hybrid film	Nitrogen oxide	100 ppm	NA	70 °C	[298]
PTh/ZnO	Nanocomposite	Liquefied petroleum	600 ppm to 2400 ppm	NA	RT	[268]
PTh/GO	Nanocomposite	Ethanol	400 ppm to 2000 ppm	Very short	RT	[270]
**MIP-based VOC sensors**						
MIP-Ag_2_S	Nanoparticles	1-butanol	100 ppm to 400 ppm	NA	NA	[279]
Au NPs/MIPs	Nanoparticles	Acetone	50 ppm to 300 ppm	3.7 ± 1 s	180 °C	[299]
Ag–LaFeO_3_	Nanoparticles	Methanol	1 ppm to 5 ppm	40 s to 47 s	175 °C	[300]
Polyaniline-fluoral-bilayer MIP	Film	Ammonia, formaldehyde	0 ppm to 1 ppm	NA	NA	[301]
MIP	Bulk	Hydroquinone, phenol, toluene, benzene, heptane	50 ppm to 400 ppm	5 s to 12 s	NA	[302]
MIP	Bulk	Hexanal, hexanoic acid, octanoic acid	5 µL + 5 µL + 5 µL	5 s	NA	[303]
MIP	Bulk	Acetaldehyde	NA	˂30 s	NA	[304]

## Data Availability

Not applicable.
